# Synthesis of Bisindole Alkaloids from the *Apocynaceae* Which Contain a Macroline or Sarpagine Unit: A Review

**DOI:** 10.3390/molecules21111525

**Published:** 2016-11-14

**Authors:** Md Toufiqur Rahman, Veera V. N. Phani Babu Tiruveedhula, James M. Cook

**Affiliations:** Department of Chemistry and Biochemistry, University of Wisconsin-Milwaukee, 3210 N. Cramer Street, Milwaukee, WI 53201, USA; mdrahman@uwm.edu (M.T.R.); tiruvee2@uwm.edu (V.V.N.P.B.T.)

**Keywords:** *Alstonia* genus, *Apocynaceae* family, sarpagine, macroline, ajmaline, bisindole alkaloids, biomimetic synthesis, partial and total synthesis, enantiospecific and regiospecific synthesis of alkaloids, bioactive indole alkaloids

## Abstract

Bisindole natural products consist of two monomeric indole alkaloid units as their obligate constituents. Bisindoles are more potent with respect to their biological activity than their corresponding monomeric units. In addition, the synthesis of bisindoles are far more challenging than the synthesis of monomeric indole alkaloids. Herein is reviewed the enantiospecific total and partial synthesis of bisindole alkaloids isolated primarily from the *Alstonia* genus of the *Apocynaceae* family. The monomeric units belong to the sarpagine, ajmaline, macroline, vobasine, and pleiocarpamine series. An up-to-date discussion of their isolation, characterization, biological activity as well as approaches to their partial and total synthesis by means of both synthetic and biosynthetic strategies are presented.

## 1. Introduction

Indole alkaloids are of extraordinary significance due to their structural diversity, medicinal properties and are an essential part of the drug discovery process (see the Merck Index for details) [1]. Their medicinal values are evident from folklore and their ethnopharmacological uses worldwide stem from ancient times [2,3,4,5]. Many of the alkaloids are marketed drugs [6], and other drugs are derived directly from natural products or they serve as lead compounds for further development [7] into clinical candidates ranging from anti-bacterial to anti-cancer agents [8,9,10,11,12,13,14,15].

The activity and structures of bisindole alkaloids have been reviewed and the number of isolated bisindole alkaloids are increasing [16,17,18,19]. This review is focused on the partial and total synthesis of bisindoles, as well as some dimeric indole alkaloids wherein at least one of the following monomeric units contains, as its obligate constituent, the sarpagine, macroline, ajmaline, vobasine, or pleiocarpamine systems. Because of the complexity of their structures, which are comprised of more than one alkaloidal partner, a convergent approach toward their synthesis is logical synthetically, as well as biosynthetically. This breaks down to synthesis of the monomeric alkaloidal units, followed by a biomimetic-type condensation to arrive at the bisindole framework. The number of isolated bisindoles has increased recently because of modern spectroscopic techniques (e.g., Mass spectrometry, ^13^C and 2D NMR, HMQC, HMBC, NOESY, and X-ray crystallography) which enable accurate structure determination on milligram quantities of the bisindole. The synthesis of these complex natural products can begin only when their structures are accurately confirmed [20]. Consequently, the synthesis of bisindole alkaloids, of course, must lag behind the synthesis of monomers which comprise them. An important approach, defined as partial synthesis, is to combine any monomeric alkaloid available, either by synthesis (including the synthesis from a natural product) or by isolation, with the other natural monomeric unit usually in a biosynthetic strategy. This process is commonly termed the biomimetic synthesis of bisindole alkaloids pioneered by Schmid, LeQuesne, Kutney, Magnus, and others.

In many studies, it has been shown that bisindole alkaloids exhibit more potent biological, pharmacological and medicinal properties than the corresponding monomers which comprise them [21,22], as mentioned above. There are many theories as to why this pattern of activity is observed but, to date, no concrete evidence as to the mechanism of action (MOA) has emerged. However, this is not surprising for one would not expect the same MOA in each case. As always one of the main hurdles in natural product related drug-discovery is that many natural products are present in very small quantities and cannot be isolated in quantities high enough to study their biological activity let alone structure-activity relationships. This situation is exacerbated in the case of bisindole alkaloids. Consequently, it is essential to develop better and easier access to bisindole alkaloids either synthetically or biosynthetically. Partial and total synthesis, thereby require equal emphasis in this regard. If one can synthesize the natural products in a stereospecific, regiospecific and enantiospecific sense via a practical synthetic route, these alkaloids could be obtained in appropriate amounts for detailed studies of their biological activity. One could then condense the mismatched pairs as well (i.e., (+)(−); (+)(+); (−)(−); (−)(+)).

The discovery of the Vinca alkaloids vinblastine and vincristine, originally isolated from Madagascar periwinkle, *Catharanthus roseus* (formerly known as *Vinca rosea*) [23,24,25,26], marked a major milestone in natural product-based drug development as these two are the first clinically used alkaloidal anti-cancer agents. Moreover, it is well known that the bisindole nature of these alkaloids is the key for the bisindoles are much more active than the monomeric alkaloids which comprise them. These bisindole (indole-indoline) alkaloids are one of the best treatment options in various types of cancers and have been successfully used in combination therapies since the early 1960s [27,28,29]. Besides, their MOA (interaction with tubuline, microtubule assembly inhibition, and mitosis inhibition) is one of the most effective treatment against cancer, which promoted rigorous research and development of vinblastine and vincristine resulting in many (derivatives of vincristine and vinblastine) clinical candidates to date [6,8,9,10,11,12,13,14,15,30,31,32,33,34,35,36]. As a consequence, these have profoundly stimulated the search and development of alkaloidal natural product based drugs.

The bisindoles (−)-accedinisine **1** and (−)-*N′*-demethylaccedinisine **2** exhibit anti-leishmanial activity, as well as moderate antibacterial activity against gram-positive *B. subtilis* [37,38]. Antimalarial activity of numerous indole alkaloids against *Palsmodium falciparum* (cerebral) malaria have been reported, many of which are bisindole alkaloids [21,39,40,41]. (−)-Macrocarpamine **3** and (+)-villalstonine **5** were the most active bisindoles isolated from *Alstonia macrophylla* against both *Entamoeba histolytica* and *Plasmodium falciparum* [21,42]. These alkaloids **3** and **5** also exhibited potent anti-cancer activity against lung cancer cell lines as well as melanoma, renal cell carcinoma, breast, and colon adrenocarcinoma [22,40]. Villalstonine **5** was also active against murine leukemia cell lines [43]. (+)-Dispegatrine **7** and its related monomer, (+)-spegatrine exhibited potent hypotensive activity, wherein the potency of the dimer was about an order of magniture greater than the monomer toward α_1_ and α_2_ adrenergic receptors [44]. (+)-Macralstonine **9** has been reported to have hypotensive activity [45,46]. In another study, by Keawpradub et al. on three *Alstonia* species from Thailand, bisindoles (+)-macralstonine **9**, (+)-*O*-acetylmacralstonine **10**, (+)-*O*-methylmacralstonine **11**, (+)-villalstonine **5**, and (−)-macrocarpamine **3**, as well as the monomeric alkaloids (+)-pleiocarpamine **14**, (−)-alstonerine **15**, and (−)-alstophylline **22** exhibited profound anti-*Plasmodial* activity [47]. It is important to note that a minor change in the structure of (+)-macralstonine **9** by conversion into the *O-*methyl ether **11** or *O*-acetyl ester **10**, profoundly increased its anti-*Plasmodial* activity [47]. This increase in activity of *O*-acetyl and *O*-methyl derivatives of macralstonine may be due to the increased lipophilicity which facilitated their transport across cell membranes. Accedinisine-related monomeric indole alkaloids (+)-pericyclivine (**92**, see later), (−)-perivine (**43**, see later) and (−)-vobasine (not shown) exhibited significant cytotoxicity against P388 and KB cells but were less active than the bisindole alkaloids [20,48]. (+)-Pleiocarpamine **14** has recently been found to form an unprecedented dimeric structure, (+)-bipleiophylline **12**, wherein two pleiocarpamine **14** units were linked together by means of an aromatic spacer (pyrocatechuic acid). This dimer was found to be very effective against drug-sensitive, vincristine resistant human KB and Jurkat cells [49]. This finding also supports the argument that bisindole alkaloids are more active than their progenitors since (+)-pleiocarpamine did not exhibit significant activity [21,22,47].

**Figure 1:**
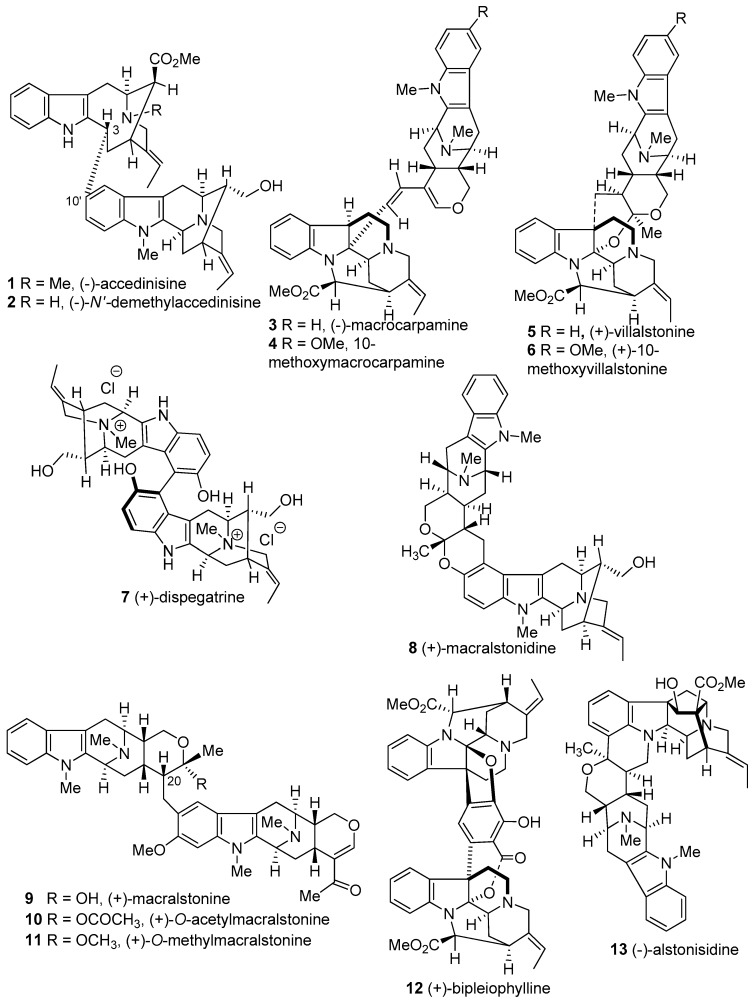
Representative examples of bioactive bisindole alkaloids.

**Figure 2:**
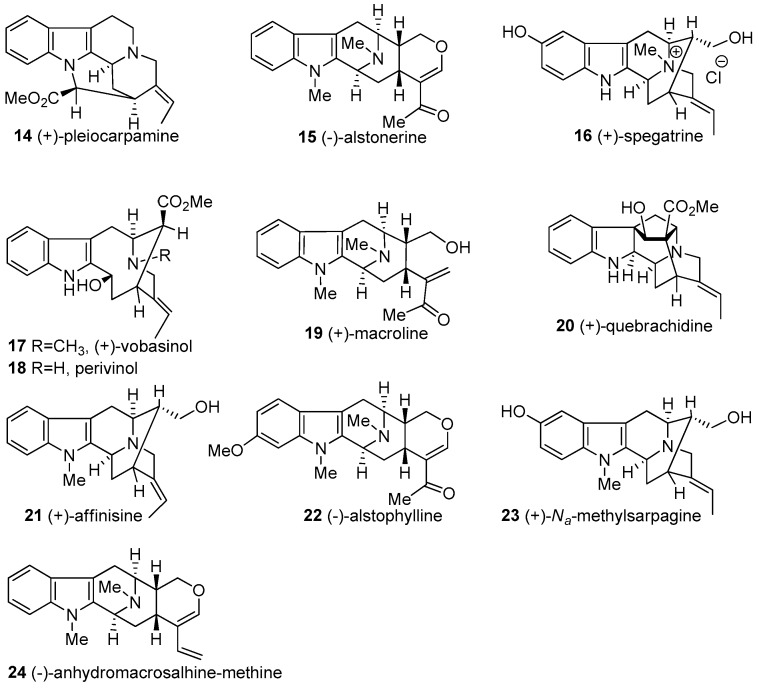
Monomeric units present in the bisindole alkaloids reviewed herein.

Illustrated in Figure 1 are representative bisindoles presented in this review. All of these alkaloids have originated from the combination of two monomeric indole alkaloids depicted in Figure 2.

## 2. Biogenetic Relationship between Sarpagine/Macroline/Ajmaline Alkaloids

**Figure 3:**
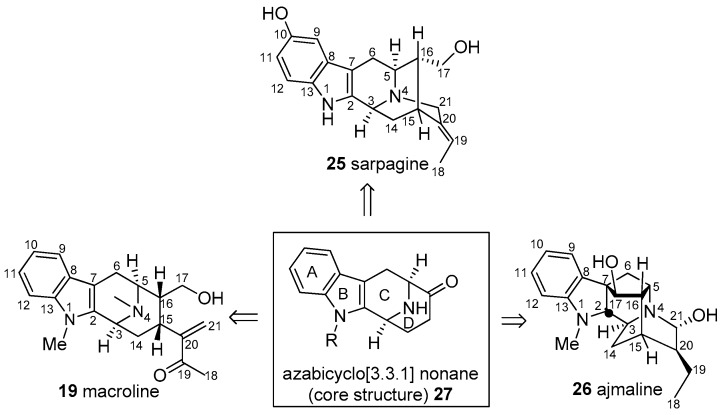
The sarpagine, macroline, and ajmaline indole alkaloids. The biogenetic numbering of LeMen and Taylor has been followed [55].

The macroline **19** and sarpagine **25** natural products are contained in one of the major classes of indole alkaloids that originated from common biogenetic intermediates. Although macroline **19** has not been isolated as an alkaloid itself, it is believed to be a biogenetic precursor for numerous alkaloids in this class. (+)-Ajmaline **26**, a clinically and historically important alkaloid [20,50,51,52], is structurally related to the sarpagine alkaloids. These three major types of indole alkaloids are inter-related through their biogenetic origin. To date, this group consists of more than 300 alkaloids which contain more than 80 bisindole alkaloids [53,54]. A general strategy has been developed to access a common intermediate to this series of natural products, the tetracyclic core of which consists of the azabicyclo[3.3.1]nonane system with the (*S*) configuration at both C-3 and C-5 (Figure 3).

**Figure 4:**
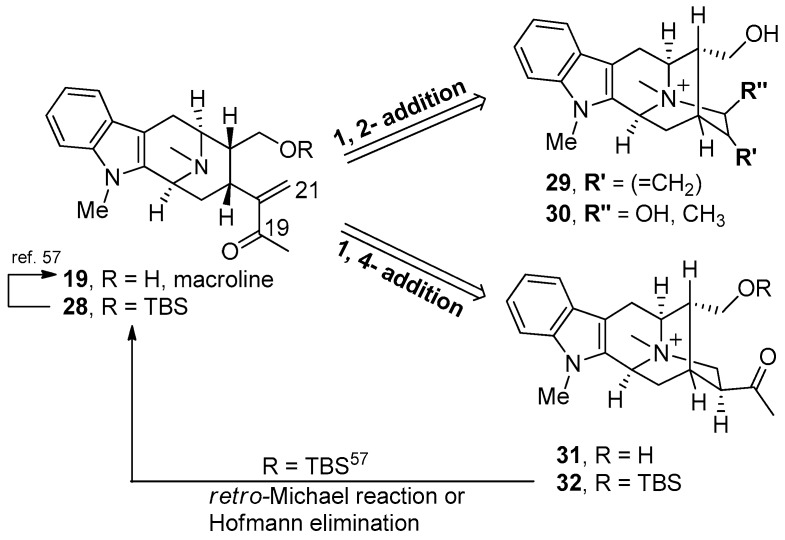
Interconversion of macroline **19** and sarpagine **25**.

In a synthetic sense, sarpagine alkaloids could originate from a Michael-type reaction of the *N*(4) nitrogen atom of macroline **19** onto the *α*,*β*-unsaturated carbonyl system at C-21 either by a 1,2 or a 1,4-type addition reaction, as illustrated in Figure 4 [54,56]. On the other hand, the sarpagine **32** can be converted into macroline **19** by a *retro*-Michael process, reported in a biomimetic sense originally by LeQuesne et al. [57].

**Figure 5:**
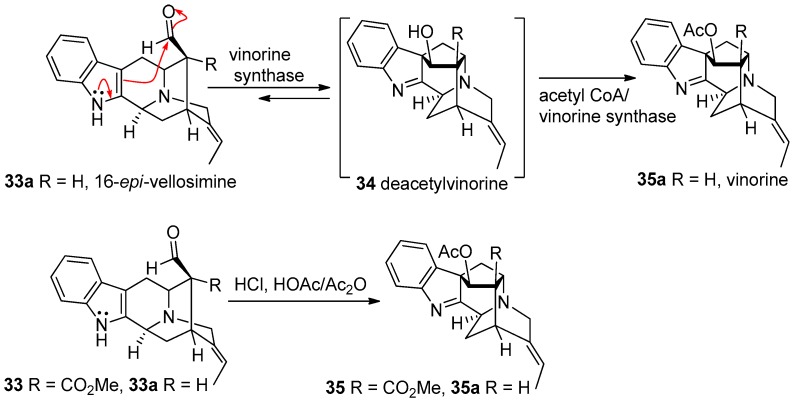
Biosynthetic relationship between sarpagine and ajmaline reported by Stӧckigt et al. [62,63,64].

The biogenetic relationship between these series of alkaloids has been reported previously [58,59,60,61]. Stӧckigt et al. converted 16-*epi*-vellosimine **33a** into vinorine **35a** via deacetylvinorine **34** using acetyl CoA dependent vinorine synthase [62,63,64]. This study confirmed the conversion of the *endo*-aldehyde containing sarpagine skeleton (**33**) into the ajmaline skeleton **(35**) under acidic conditions, as suggested earlier by Woodward (Figure 5) [58].

## 3. General Strategy to Access the Tetracyclic Core 27 of the Sarpagine/Macroline/Ajmaline Related Indole Alkaloids

An azabicyclo[3.3.1]nonane core system **27** is a common feature of the macroline/sarpagine/ajmaline type indole alakaloids. Thus, a general synthetic strategy for the synthesis of these alkaloids involved the synthesis of this core structure with the correct stereochemistry at positions C-3, C-5 and appropriate functional groups at C-15 and C-16 for further functionalization. To date, methods for accessing the (±)-azabicyclo[3.3.1]nonane **27** system has been reported by Yoneda [65], Mashimo [66], Kluge [67] and on large scale by Soerens et al. [68].

**Scheme 1:**
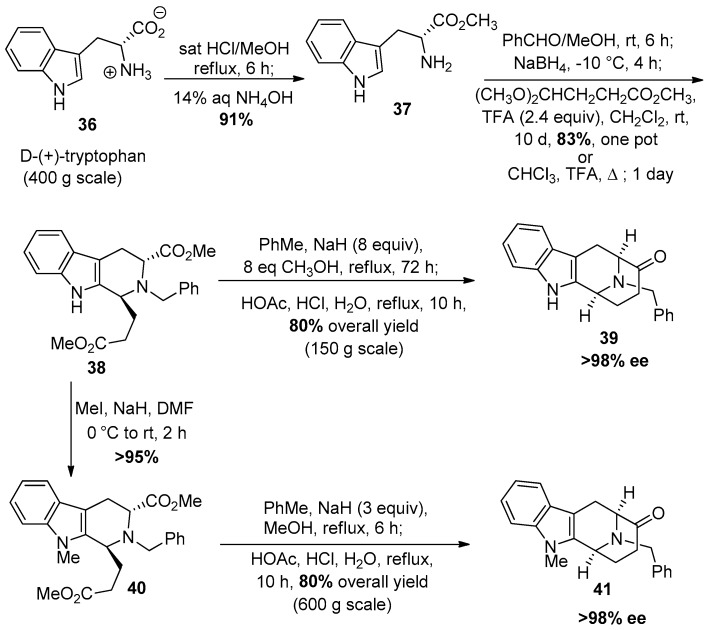
General strategy to access the tetracyclic ketones which contain the azabicyclo[3.3.1]nonane system.

The synthesis of the optically active (−)-tetracyclic core was achieved by Zhang et al. [69] in 1988 in a stereospecific fashion which employed the asymmetric Pictet-Spengler reaction. Numerous adjustments have been made to better access this moiety with both *N*_a_-H and *N*_a_-Me functionalities over the years. The present strategy is illustrated in Scheme 1. Currently, the Pictet-Spengler reaction is carried out in one pot under conditions that permit epimerization at C-1 to provide only the desired *trans*-diastereomer **38**. d-(+)-tryptophan methyl ester **37** is commercially available but can also be prepared from d-(+)-tryptophan **36** by Fischer esterification on 400 gram scale.

The *N*_b_-benzylation was done on amine **37** with benzaldehyde in methanol at room temperature, followed by reduction of the so formed iminium ion salt with sodium borohydride added slowly to the mixture at −10 °C. Subsequently, trifluoroacectic acid was added to the mixture at 0 °C to bring the pH to 7. After removal of the solvent, methylene chloride, trifluoroacetic acid (TFA) and 4,4-dimethoxybutyrate were added to the mixture at 0 °C and this was stirred for 10 days at room temperature to yield the *trans*-diester **38** in 83% yield as the sole product [70]. This could be accomplished in refluxing chloroform in one day in slightly lower yield. Next, the Dieckmann cyclization was done in the presence of a large excess of NaOMe and methanol in toluene at reflux for 72 h (6 h for the *N*_a_-Me indole) to yield the cyclized product. At the completion of the reaction, the reaction mixture was cooled to 0 °C and the excess base was neutralized with glacial acetic acid and the solvent was removed under reduced pressure. After that, glacial acetic acid and aqueous hydrochloric acid as well as water were added to the mixture and it was allowed to reflux under these strongly acidic conditions to furnish the product of decarboxylation. This gave *N*_b_-benzyl tetracyclic ketone **39**, in 80% overall yield on large scale. For the *N*_a_-Me series, the *trans*-diester **38** was methylated using iodomethane and sodium hydride in DMF to yield the *N*_a_-Me *trans*-diester **40** in >95% yield, which under the same conditions as **38** gave the *N*_a_-Me tetracyclic ketone **41** in a one-pot process. This route is still one of the best routes since reactions can be done on multi-hundred gram scales and do not require protection of the *N*_a_-H function. This is an enantioselective and regioselective process in >98% ee. This route with two one pot conversions also requires fewer steps than previous routes [69,70,71]. In addition, the availability of d-(+)-tryptophan and the (−)-enantiomer provides a route to both enantiomers of the alkaloids including the ability to condense mismatched pairs, as mentioned.

## 4. Partial and Total Synthesis of Bisindole Alkaloids

### 4.1. (−)-Alstonisidine

(−)-Alstonisidine was isolated from the bark of *Alstonia muelleriana* Domin, along with several other alkaloids by Elderfield et al. and LeQuesne et al. [72,73]. The structure of (−)-alstonisidine **13** was reported in 1972 by LeQuesne et al. [74,75]

**Figure 6:**
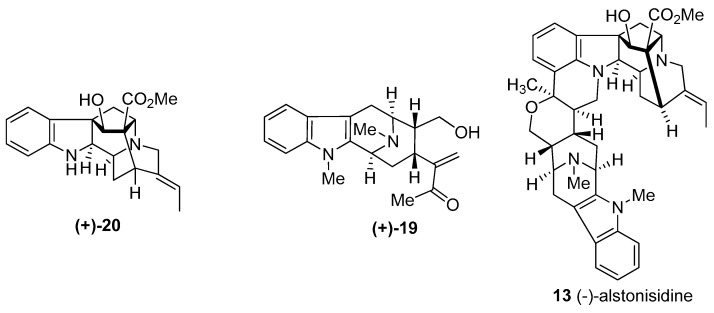
Structures of (+)-quebachidine (**20**), (+)-macroline (**19**), and (−)-alstonisidine (**13**).

The structure of **13** was proposed, in a biomimetic sense, for (−)-alstonisidine from its constituent monomeric units, (+)-quebrachidine **20** and (+)-macroline **19**. The crystal structure of (−)-alstonisidine was ultimately reported by Hoard in 1977 which confirmed the structure as **13** (Figure 6) [76]. When the mixture of **19** and **20** was stirred in 0.2 *N* aqueous HCl, the labile hemiketal **42** formed, as indicated by examination of the spectral data. The tentative stereochemistry was based on the structure of the bulky quebrachidine unit which bore a –CH_2_– group which should occupy the equatorial position. The hemiketal was treated with boron trifluoride etherate to yield alstonisidine which suggested the structure **42** as an intermediate, since no reaction occurred under the same conditions between (+)-quebrachidine **20** and (+)-macroline **19** [75].

This biomimetic synthesis of (−)-alstonisidine **13** employed the biogenetic precursors (+)-macroline **19** and (+)-quebrachidine **20**. Although macroline has never been isolated as a natural product, it is widely agreed to be a biogenetic precursor for (−)-alstonerine [77], (−)-alstophylline [78], and (+)-alstonisine [72] and may be present in very small steady-state concentrations in plants. Furthermore, the isolation of traces of (+)-quebrachidine from *A. muelleriana* further strengthened this proposed biogenetic route. (+)-Quebrachidine **20** has been isolated from *Vinca libanotica* [79], *Ochrosia acuminate* [80], *Alstonia muelleriana* [77], and the leaves of *Aspidosperma quebracho blanco* Schletch [81] and its structure was proposed to be **20** based on the studies by NMR and mass spectroscopy [82]. Four aromatic proton multiplets (δ = 6.6 to 7.2) indicated it contained an unsubstituted aromatic ring, accompanied by an ethylidene group. Examination of the Infrared spectrum indicated the presence of *N*-H (3590 cm^−1^), *O*-H (3370 cm^−1^) and a carbomethoxyl (1722, 1235 cm^−1^ and ^1^H singlet at δ = 3.6) group. Upon acetylation, the compound gave a diacetate. Analysis of the NMR, IR and UV spectra of the diacetate indicated the presence of a secondary alcohol and a dihydroindole nitrogen atom. Analysis of the mass spectrum further strengthened the proposed structure of (+)-quebrachidine to be **20** [82].

**Scheme 2:**
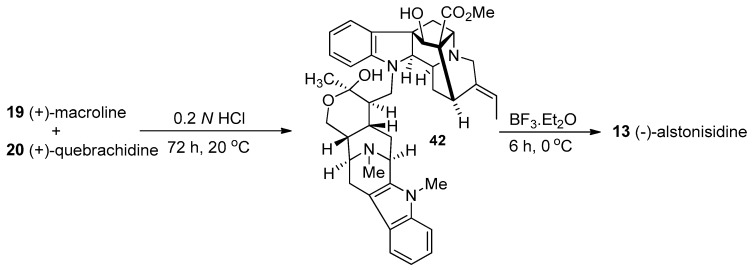
Biomimetic partial synthesis of (−)-**13** by LeQuesne et al. [75].

In the biomimetic partial synthesis of (−)-alstonisidine **13** [83], (+)-macroline **19** was prepared by degradation of villamine (not shown) by the method of Schmid et al. [84]. (+)-Macroline **19** reacted with naturally occurring (+)-quebrachidine **20** under mildly acidic conditions at 20 °C (Scheme 2). Initially macroline underwent a Michael reaction with the indoline nitrogen atom of quebrachidine and subsequently underwent intramolecular cyclization to form the amino hemiketal **42**. This hemiketal species was found to be highly labile to acid since it reversed to the starting materials **19** and **20** upon warming, as well as under stronger acidic conditions and during chromatography. Fortunately, no other compounds were observed except the starting materials **19** and **20**. The new indole **42** had a molecular weight of 690 and examination of the IR spectrum indicated the presence of only one carbonyl peak (1735 cm^−1^) which indicated that after the initial 1,4-conjugate addition had occured, the so formed ketone on the macroline unit reacted intramolecularly with the –CH_2_OH group to form the corresponding hemi-ketal present in **42**. When hemiketal **42** was treated with boron trifluoride at 0 °C for 6 h, the electrophilic aromatic substitution took place, *ortho* to the amino group of quebrachidine to form the tetrahydropyran which was indistinguishable from the naturally occurring alstonisidine **13** (Scheme 2) from *A. muelleriana* [77,83].

The total synthesis of (−)-alstonisidine has not been reported yet. Extensive work has been done toward its monomeric constituents **19** and **20**. To date, The Milwaukee group has achieved the total synthesis of (+)-macroline **19** [85,86,87,88] and the enantiomer of macroline as well [89]. Besides an advanced intermediate for **20**, the synthesis of quebrachidine diol (not shown) has been reported as well [90]. The partial synthesis of (−)-alstonisidine has been completed, to date, from synthetic (+)-macroline **19** and natural (+)-quebrachidine **20**. The total synthesis of bisindole **13** awaits the total synthesis of **20**.

**Scheme 3:**
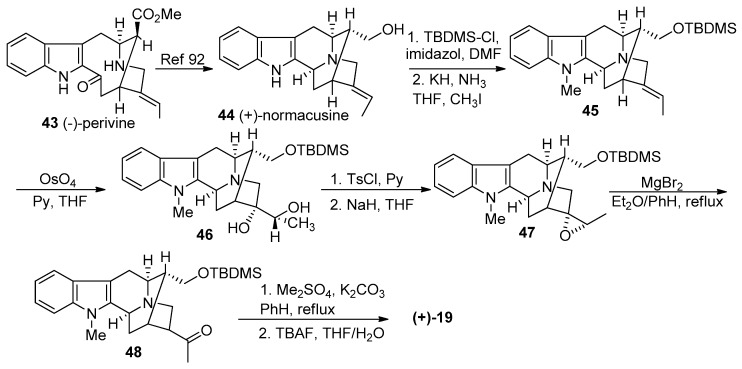
Biomimetic partial synthesis of (+)-**19** by LeQuesne et al. [57].

The earliest synthesis of (+)-macroline **19**, a biomimetic synthesis, was done by LeQuesne et al. in 1980 [57] and the absolute configuration had been confirmed by X-ray crystallography by Hesse in 1981 [91]. In this synthesis, LeQuesne et al. converted (−)-perivine **43** to (+)-normacusine **44**, according to the reported procedure by Gorman et al. (Scheme 3) [92]. The protection of the hydroxyl group (in **44**) as the *tert*-butyldimethylsilyl ether, was followed by *N*_a_-methylation to furnish the sarpagine system **45**. Osmylation of olefin **45** with OsO_4_ and pyridine in THF gave an inseparable mixture which contained the diol **46**, along with a related spirooxindole byproduct. The bulky TBDMS function rendered the osmylation sequence stereoselective, although marred by the formation of the spirooxindole side-product. This mixture was treated with TsCl and Py, followed by NaH in THF to give the epoxide **47** that could be purified by crystallization. The epoxide underwent rearrangement on treatment with freshly prepared MgBr_2_ to furnish the ketone **48**. The methylation of the *N*_b_ function was performed with dimethyl sulfate and potassium carbonate, in refluxing benzene. This was followed by the *retro*-Michael reaction in base followed by desilylation with TBAF to provide (+)-macroline **19** [57].

**Scheme 4:**
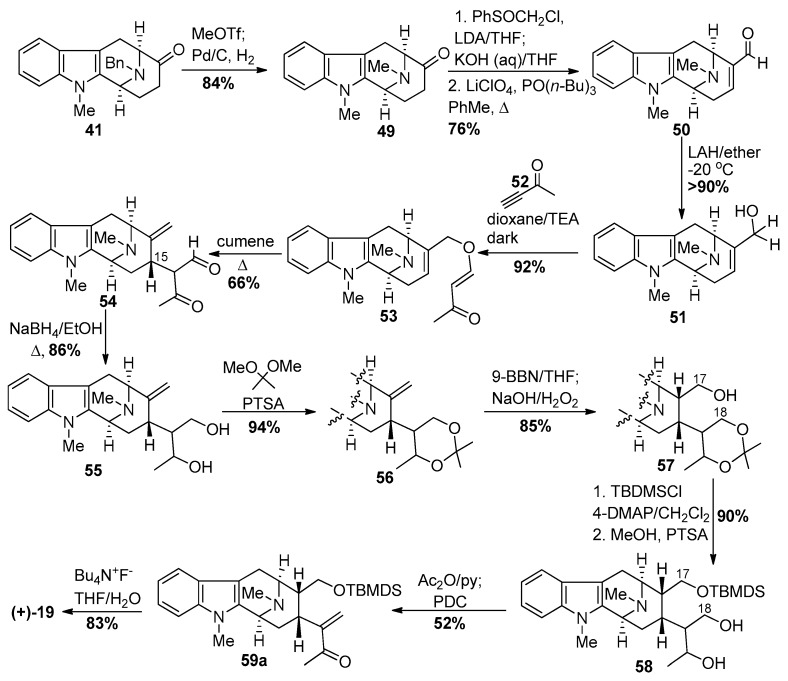
Enantiospecific total synthesis of (+)-**19** by Bi et al. [85].

In an enantiospecific synthetic approach to (+)-macroline [85,86], the readily available *N*_b_-benzyl tetracyclic ketone [93,94,95] intermediate **41**, available on 300 gram scale, was converted into the corresponding *N*_b_-methyl indole compound **49** by methylation with methyl triflate followed by catalytic debenzylation to form the *N*_b_-methyl system in 84% yield (Scheme 4). The ketone **49** was reacted with the anion of chloromethyl phenyl sulfoxide (LDA, THF, −78 °C) to generate the corresponding chlorohydrin. This chlorohydrin, upon stirring in a heterogeneous solution of 10 *N* KOH in THF at 25 °C for 24 h produced the corresponding spirooxirane and this was followed by treatment with LiClO_4_ and PO(*n*-Bu)_3_ in refluxing toluene to produce the *α*,*β*-unsaturated aldehyde **50** [96]. Chemo-selective reduction of the aldehyde with lithium aluminum hydride in ether at −20 °C gave the allylic alcohol **51**. Michael addition of the alcohol to 3-butyn-2-one **52** provided the desired enone ether **53** in 92% yield. The Claisen rearrangement of the enone ether produced the *β*-dicarbonyl compound **54**, which was subsequently reduced with sodium borohydride to give the corresponding diol **55**. The 1,3-diol functionality was protected as an acetonide **56** using acetone dimethyl acetal in the presence of *p*-toluenesulfonic acid. The hydroboration of the olefin in **56** with 9-BBN occurred in a stereospecific fashion and this was followed by oxidation with a basic solution of hydrogen peroxide, under classical conditions, to furnish the C-17 primary alcohol **57**. The hydroxyl group of monol **57** was protected as the *t*-butyldimethylsilyl ether, and subsequent selective removal of the acetonide was done using *p*-toluenesulfonic acid in dry methanol to provide diol **58**. To access the *α*,*β*-unsaturated carbonyl system in macroline, it was planned to use the tosylate of the C-18 hydroxyl group as the leaving group. But the tosylation with tosyl chloride and pyridine in DCM was not successful, and mesylation took place with very poor regioselectivity. Based upon a precedent by Helquist et al. [97], acetylation with acetic anhydride worked both as a protecting group and leaving group. The so formed monoacetate was subjected to PDC oxidation. The acetate group underwent elimination spontaneously upon oxidation to provide the stable TBDMS protected macroline equivalent **59a**, which could be stored in a vial. The silyl ether was deprotected with tetrabutylammonium fluoride in THF to provide (+)-macroline **19** identical to the natural material obtained from villamine by Schmid et al. [84].

**Scheme 5:**
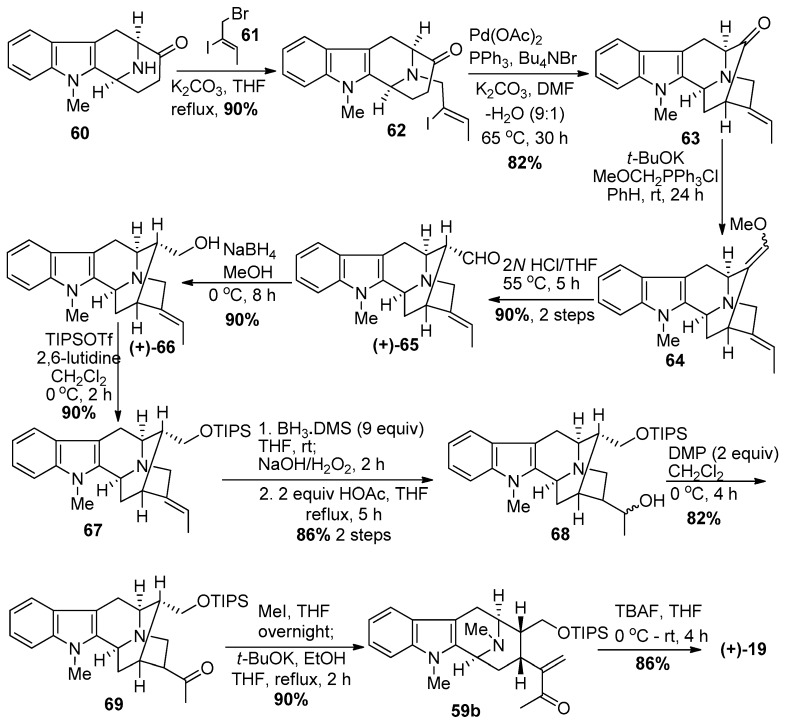
Improved total synthesis of (+)-macroline (**19**) by Liao et al. [87].

In another more recent and improved approach (Scheme 5) [87,88], *N*_b_-alkylation via S_N_2 substitution on (*Z*)-1-bromo-2-iodo-2-butene **61** in the presence of K_2_CO_3_ in refluxing THF gave the vinyl iodide **62**. The palladium catalyzed intramolecular cyclization of the enolate anion with the vinyl iodide took place in a stereospecific manner to yield the pentacyclic ketone **63**. A one-carbon homologation via Wittig olefination with methoxymethyl triphenylphosphonium chloride with potassium *tert*-butoxide in benzene, followed by hydrolysis of the so formed enol ether **64** in aqueous HCl/THF furnished the *α*-aldehyde **65** ((+)-*N*_a_-methylvellosimine; an alkaloid isolated from *Alstonia nitida* [98]). The aldehyde was reduced with sodium borohydride to provide (+)-affinisine **66**, another alkaloid obtained from *A. macrophylla* [99]. The protection of the primary alcohol as the TIPS ether with triisopropylsilyl triflate and 2,6-lutidine gave indole **67**. The hydroboration-oxidation of the olefin in **67** went smoothly to provide the secondary alcohol **68** in very good yield, accompanied by a small amount of the corresponding tertiary alcohol. An equivalent of BH_3_ is known to complex with the *N_b_*-nitrogen function which alters the electronic character in the vicinity of the olefin, which presumably resulted in the formation of the small amount of tertiary alcohol. The Dess-Martin periodinane-mediated oxidation of the alcohol **68** to the ketone **69** took place in high yield. The quaternization of the *N*_b_-nitrogen with methyl iodide in THF at 0 °C, was followed by treatment of the so formed *N*_b_-methiodide salt with potassium *tert*-butoxide in ethanol under modified conditions to provide the stable macroline equivalent **59b**. Again, this intermediate can be easily stored. The de-silylation with tetrabutylammonium fluoride resulted in the synthesis of (+)-macroline **19** identical to that obtained above in much higher overall yield and fewer steps. These reactions could be scaled up with ease as well.

**Scheme 6:**
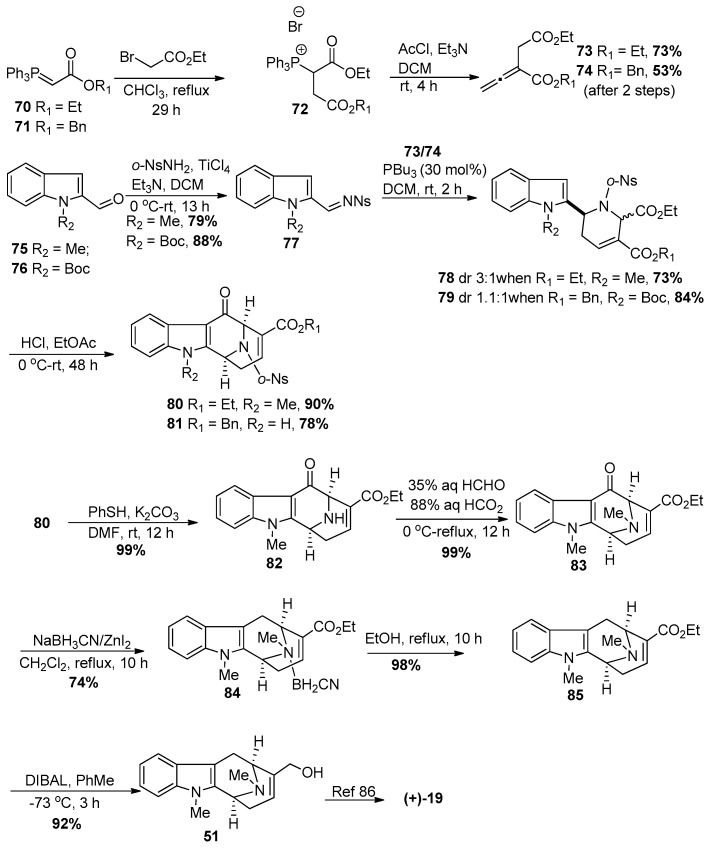
Formal synthesis of (+)-macroline (**19**) by Kwon et al. [100].

Kwon recently reported a formal synthesis of (+)-macroline **19** as well, which employed a phosphine-catalyzed [4 + 2] annulation process (Scheme 6) [100]. This route began from commercially available phosphorene **70** or **71**; the first step of which was to generate the phosphonium salt **72** by reaction with the bromoacetate. Treatment of **72** with acetyl chloride in the presence of trimethylamine furnished the allene **73** or **74**, individually. The condensation of *o*-nitrobenzenesulfonamide with either of the indole-2-carboxaldehydes **75** or **76** in the presence of TiCl_4_ and Et_3_N gave the imines **77** in very good yield, respectively. The allene (**73** or **74**) was then reacted with the imine **77** in the presence of tributylphosphine in methylene chloride to yield the [4 + 2] annulation products as inseparable mixtures with moderate diastereoselctivity (dr 3:1 when R_1_ = Et, R_2_ = Me and dr 1.1:1 when R_1_ = Bn, R_2_ = Boc). The C-5 carboxylic acid ester was epimerized under acidic conditions to facilitate the intramolecular Friedel-Crafts acylation to provide the bridged bicyclic compounds **80** or **81**.

The nosyl group on the *N*_b_-nitrogen atom in **80** was removed by the method of Fukuyama [101] and proceeded smoothly to provide the *N*_b_-H free base **82** in excellent yield (Scheme 6). An Eschweiler-Clarke reaction [102,103] gave the otherwise troublesome *N*_b_-methylated product **83** in quantitative yield. The deoxygenation at C-6 under standard reductive conditions either did not react or gave the C-6 alcohol. However, the use of ZnI_2_-NaCNBH_3_, gave the desired aryl alkane as an *N*_b_-complex **84** with cyanoborane, which upon heating in refluxing ethanol gave the free amine **85**. Reduction of **85** with DIBAL in toluene gave the allylic alcohol **51**, a known intermediate toward (+)-macroline **19** and (−)-alstonerine **15** previously reported [86] by The Milwaukee group and hence this completed a formal synthesis of (+)-macroline **19**.

### 4.2. (−)-Accedinisine and (−)-N′-demethylaccedinisine

**Scheme 7:**
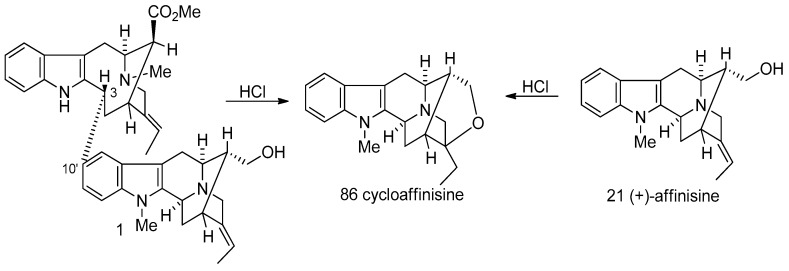
Formation of cycloaffinisine from (−)-accedinisine (**1**) and (+)-affinisine (**21**) under acidic conditions.

(−)-Accedinisine **1** was isolated along with several other bisindole alkaloids from *Tabernaemontana accedens* Muell. Arg. (Apocynaceae) and *Peschiera van heurkii* [38,104]. This dimeric indole alkaloid contained both a vobasine and a sarpagine unit. Upon treatment with hydrochloric acid, natural accedinisine **1** yielded cycloaffinisine **86** which could also be prepared from affinisine **21** under the same conditions. This clearly indicated the presence of an affinisine **21** unit in this dimeric alkaloid. Extensive studies of **1** by NMR and mass spectroscopy of the bisindole suggested the presence of a vobasinol unit as the remaining part of the molecule which was confirmed by the condensation of (+)-vobasinol **17** with (+)-affinisine **21** in aqueous HCl to yield a compound identical to natural (−)-accedinisine **1** (Scheme 7) [104]. Another vobasine-sarpagine (perivinol **18** + affinisine **21**) bisindole alkaloid (−)-*N′*-demethylaccedinisine **2** (demethylaccedinisine) was isolated from *Peschiera buchtieni* [105] and *Peschiera van heurkii* [38]. The structure was confirmed by methylation of (−)-**2** to yield the identical compound to the authentic sample of (−)-accedinisine **1** [105]. Both of this dimeric indole alkaloids have the constituent monomeric alkaloids linked from the C-3 position of the vobasine unit (vobasinol **17** or perivinol **18**) to the C-10′ of the sarpagine unit (affinisine **21**), as indicated by NMR spectroscopy and partial synthesis [104,105]. Both of these bisindoles are active against leishmaniasis. It is of interest that these alkaloids are purportedly active against only one of the strains of leishmaniasis that were studied indicating selectivity.

**Scheme 8:**
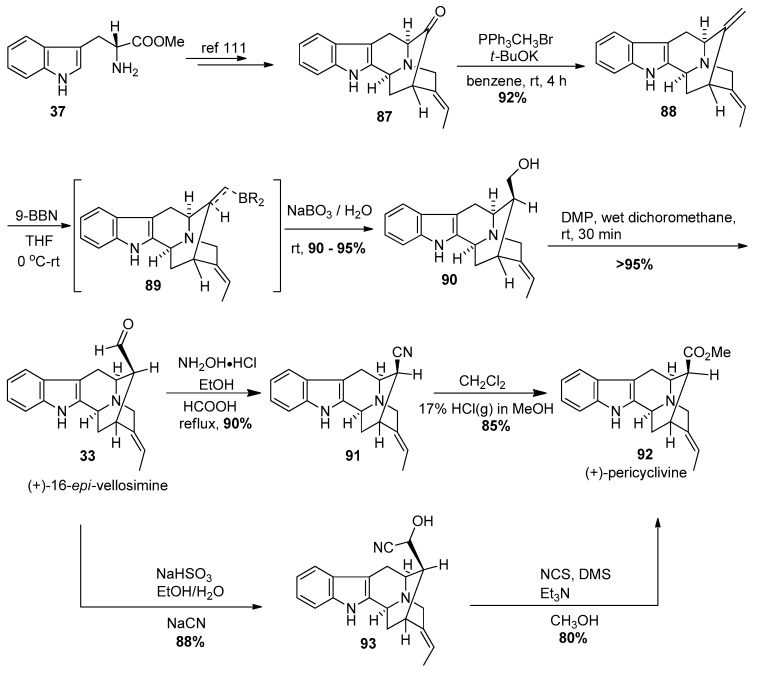
Enantiospecific total synthesis of (+)-16-*epi*-vellosimine (**33**) and (+)-pericyclivine (**92**) by Yang et al. [106,107].

The vobasine portion of these alkaloids originated from C(3)-*N*_b_ disconnection of the sarpagine skeleton of (+)-peicyclivine (see later) and similar to the other unit, (+)-affinisine **21**, could be accessed by a general and efficient approach from commercially available d-(+)-tryptophan methyl ester **37** (Scheme 8). Yang et al. achieved a total synthesis of (−)-accedinisine **1** and (−)-*N′*-demethylaccedinisine **2** [106,107,108,109].

The synthesis of the northern vobasine fragment was accomplished from the previously synthesized (+)-16-*epi*-vellosimine (from d-(+)-tryptophan [110]). The *E*-ethylidene ketone **87** was available in an enantiospecific manner in five steps starting from commercially available d-(+)-tryptophan (Scheme 8) [111]. The Wittig olefination with methyltriphenylphosphonium bromide in the presence of potassium *tert*-butoxide in benzene provided the olefin **88** in excellent yield. Improved access to the aldehyde from the olefin via hydroboration with 9-BBN was followed by Kabalka oxidation with NaBO_3_·4H_2_O to furnish (+)-16-*epi*-normacusine **90** in excellent yield. This was followed by Dess-Martin oxidation to yield the *β*-aldehyde, in (+)-16-*epi*-vellosimine **33** in >95% yield in the absence of any epimerization. The aldehyde was treated with hydroxylamine hydrochloride and subsequesnt dehydration of the so formed oxime which resulted in the corresponding cyano-compound **91** in 90% yield. The cyano function was converted into the corresponding methyl ester, (+)-pericyclivine **92**, under the conditions (HCl(g), CH_2_Cl_2_, CH_3_OH) reported by Warmuth et al. [112]. In an alternative approach, (+)-16-*epi*-vellosimine **33** was converted into (+)-pericyclivine via a cyanohydrin that resulted from nucleophilic attack of the cyano group onto the bisulfite adduct of 16-*epi*-vellosimine analogous to a previous report by Wender et al. [113]. The cyanohydrin **93** was oxidized to the ketone at low temperature under Corey-Kim oxidation conditions, which yielded the ester (+)-pericyclivine **92** in 80% yield, on treatment with methanol. This constituted the first enantiospecific total synthesis of the indole alkaloid (+)-pericylivine.

**Scheme 9:**
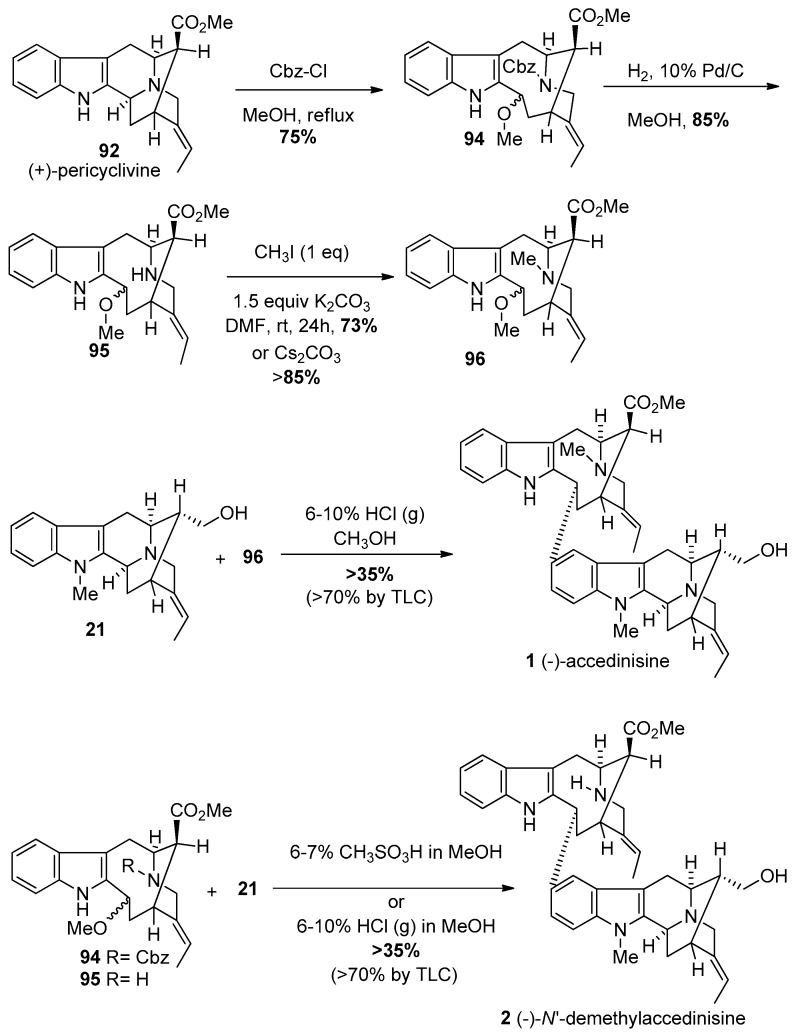
Completion of the total synthesis of bisindole alkaloids (−)-**1** and (−)-**2**.

The C(3)-*N*_b_ bond cleavage [114,115,116,117,118,119,120,121] was carried out analogous to the procedure reported by Bonjoch et al. [114]. Pericyclivine **92** when heated to reflux in methanol, and reacted with benzyl chloroformate, to furnish the carbamate **94** in 75% yield (Scheme 9). The Cbz-group was removed by standard hydrogenolysis with hydrogen and palladium on carbon in methanol. This was followed by methylation of the *N*_b_-group to provide the vobasine unit **96** required for (−)-accedinisine. The key to this synthesis was the formation of the 9-membered ring in **94** for the *pseudo*-axial ester and the *pseudo*-equatorial ester are similar in energy so there is no driving force for epimerization. This was in contrast to the *β*-ester function in **92**, which can undergo epimerization to the *α*-configuration upon chromatography on silica gel.

(+)-Affinisine **21** is a sarpagine-type indole alkaloid isolated from *Alstonia macrophylla* [99] Wall, *Peschiera affinis* [122], and *Ervatamia hirta* [123]. In 2000, Liu et al. reported an enatiospecific total synthesis of the enatiomer of affnisine from l-(−)-tryptophan [124]. In 2006, Liao et al. completed the total synthesis of (+)-affinisine as an intermediate in the total synthesis of (+)-macroline and (−)-alstonerine [87].

The *N*_b_-Cbz derivative **94** and *N*_b_-methyl derivative **96** provided the northern hemispheres of (−)-*N′*-demethyl accedinisine **2** and (−)-accedinisine **1**, respectively. The coupling of (+)-affininsine **21** with *N*_b_-methyl intermediate **96** in 6%–7% methanolic HCl or with *N*_b_-Cbz intermediate **94** in 6%–7% methanolic methanesulfonic acid or HCl(g) [125,126] provided the corresponding bisindoles in stereospecific fashion with an un-optimized yield of >35% (Scheme 9). There was one major component on TLC (silica gel) with a trace of a material too little to isolate with no starting materials left and no baseline material. Both of these dimers were run on milligram scale and were purified by preparative TLC on an actual thin layer plastic backed plate. The recovery was very poor. It is clear only one diastereomer was formed and from TLC the yield of the bisindoles appeared to be greater than 70%.

### 4.3. (+)-Macralstonine

**Figure 7:**
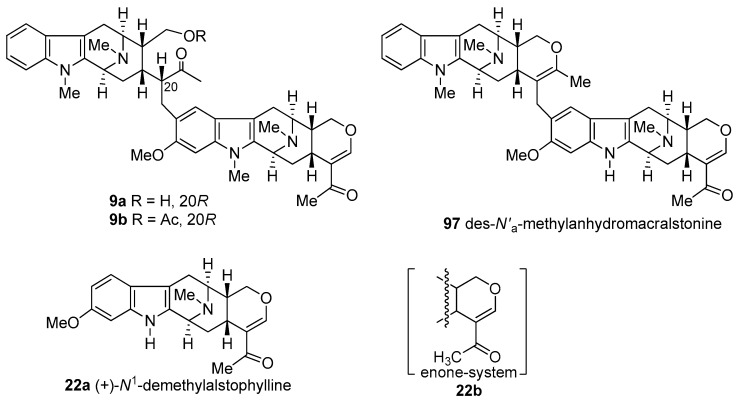
Structures of the cyclic ketone form (**9a**) of (+)-macralstonine and its *O*-acetyl derivative (**9b**); des-*N′*_a_-methylanhydromacralstonine (**97**) as well *N*^1^-demethylalstophylline (**22a**), and the enone system (**22b**).

(+)-Macralstonine **9** is a dimeric alkaloid which consists of two macroline cores which belong to the *Alstonia* alkaloids (−)-alstophylline **22** and (+)-macroline **19**. This bisindole was isolated from *Alstonia macrophylla* [22,127,128,129,130], *Alstonia angustifolia* [131], *Alstonia muelleriana* [132], and *Alstonia glabriflora* Mgf. [133]. Several related bisindoles (natural or semi-synthetic) with potent antimalarial activity, (+)-*O-*methylmacralstonine [22], (+)-*O*-acetylmacralstonine [40], and (+)-des-*N*′_a_-methylanhydromacralstonine **97** [73] were also known. In their studies, Schmid et al. [128] reported that (+)-macralstonine underwent ring opening and existed in an equilibrium mixture of the acyclic ketone **9a** (Figure 7) and the cyclic hemiketal **9** in chloroform solution. This has been confirmed recently by Kam et al. by the analysis of high field NMR (600 MHz) spectroscopy that the ratio of acyclic to cyclic macralstonine was 2.32:1, 1.14:1, and 0:1 in CDCl_3_, CD_2_Cl_2_, and THF-*d*_8_ respectively. In their NMR (NOESY) spectroscopic studies of the *O-*methyl congener **11** during its isolation from *A. macrophylla* [22,130], Keawpradub et al. and Changwichit et al. concluded the stereochemistry at C-20 to be *R* (*β*-H). In the case of macralstonine [129], NOESY was not feasible. Fortunately, it was possible to trap the acyclic form as the *O-*acetyl derivative **9b** (Figure 7) which clearly indicated the C-20 to be in *R* (*β*-H) configuration after spectroscopic analysis. This was further confirmed by X-ray analysis of (+)-macralstonine which was crystallized as the hemiketal **9** [129].

**Scheme 10:**
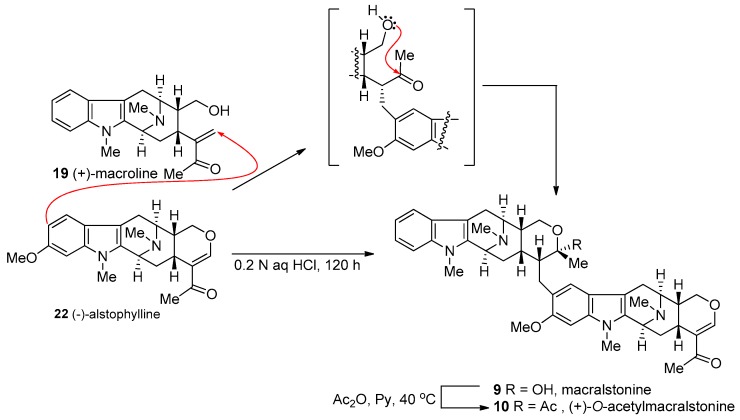
The biomimetic partial synthesis of macralstonine (**9**) and its *O*-acetyl derivative (**10**) by LeQuesne et al. [135].

One possible biosynthetic route to (+)-macralstonine **9** was proposed by Schmid [134], which was confirmed in a biomimetic partial synthesis by LeQuesne et al. [135] by coupling (+)-macroline **19** with (−)-alstophylline **22** in dilute 0.2 *N* aqueous HCl solution (Scheme 10).

This 0.2 *N* aqueous hydrochloric acid solution which contained alstophylline and excess (+)-macroline was stirred at 20 °C for 5 days. (+)-Macralstonine **9** was isolated in 40% yield after chromatographic separation. Examination of the product by thin layer chromatography and infrared spectroscopy indicated this material was identical to the natural (+)-macralstonine and the analysis of the ^1^H NMR spectrum indicated the alkaloid was a mixture of C-20 epimers. Upon acetylation with actetic anhydride in pyridine, the C-20 epimer of macralstonine underwent epimerization to provide only (+)-*O*-acetylmacralstonine **10** indistinguishable from the acetyl derivative of natural (+)-macralstonine (Scheme 10) [40].

**Figure 8:**
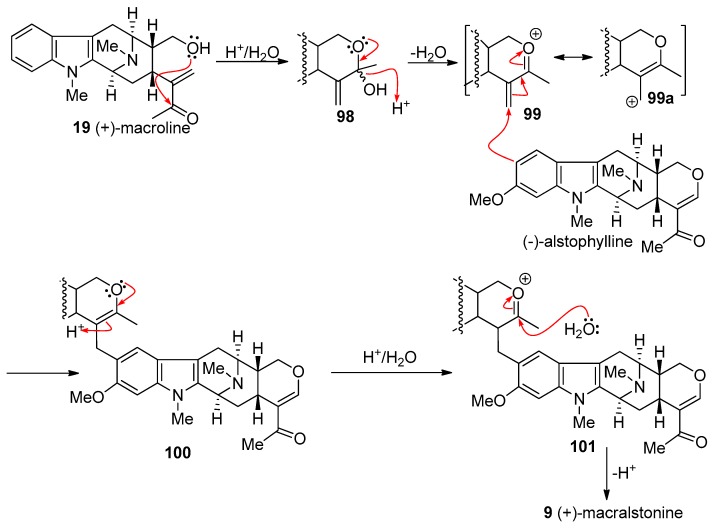
The possible alternative mechanism for the formation of (+)-**9** via a Friedel-Crafts alkylation process.

At least two mechanisms are possible for the formation of macralstonine **9** by the acid catalyzed coupling of macroline and the ring-A oxygenated macroline-type indole base (−)-alstophylline **22**. One possible mechanism was the earlier described Michael process [135]; however another potential mechanism is a Friedel-Crafts alkylation process stabilized by the oxonium ion [136]. The Michael addition followed by cyclization is straight-forward, as shown in Scheme 10. The electron-rich aromatic ring adds nucleophiliclly in a 1,4-fashion to form the alkylated product *ortho* to the methoxy group, followed by nucleophilic addition of the C-17 hydroxy group to the carbonyl carbon atom to form the cyclic hemiketal present in macralstonine **9**. On the other hand, alternatively, acid catalyzed intra-molecular cyclization of (+)-macroline, which involves the nucleophilic attack of the C-17 hydroxyl group on to the carbonyl carbon atom (1,2-fashion) would provide the cyclic hemiketal **98**. This intermediate can then lose a molecule of water to form the oxonium ion species **99** (Figure 8). Electrophilic attack of the electron rich aromatic ring onto the oxonium ion electrophile would furnish the cyclic enol-ether **100**, as suggested by Fukuyama. The cyclic enol-ether would then isomerize to an oxonium ion, only to be attacked by water to provide the cyclic hemiketal present in macralstonine **9**.

(−)-Alstophylline **22**, as described above comprises one unit of the antimalarial bisindoles macralstonine and the related *O*-acetyl and *O*-methyl macralstonine bases. This alkaloid was originally isolated from *Alstonia macrophylla* WALL by Kishi, Schmid et al. [78,128], from *Alstonia glabriflora* Mgf. by Hart [133] and from *Alstonia angustifolia* by Ghedira et al. [131]. In addition, the related *N*_a_-H analog (+)-*N*^1^-demethylalstophylline **22a** has recently been isolated from *Alstonia macrophylla* by Kam [43].

The total synthesis of (−)-alstophylline required facile access to the optically active 6-methoxy tryptophan **105** which was no easy task. It was developed by Huang, Wearing, Liu et al. [137,138] on large scale. In the initial approach towards several 11-methoxy macroline-related indole alkaloids, Liu et al. completed a total synthesis of (−)-alstophylline [139].

**Scheme 11:**
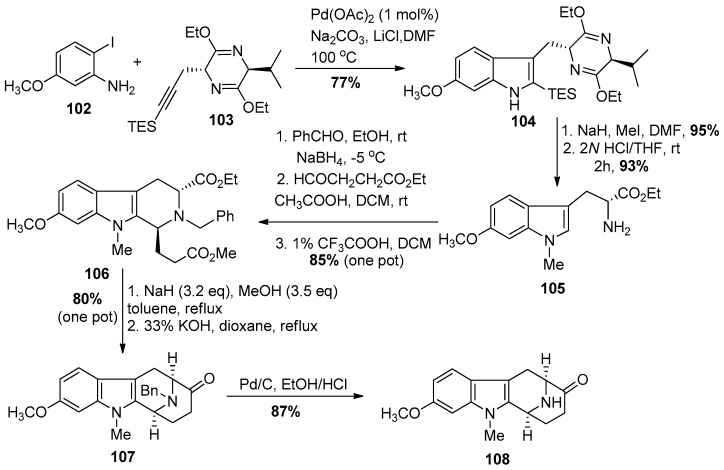
Large scale enantiospecific access to the 11-methoxy substituted tetracyclic core structure present in (−)-alstophylline (**22**).

The readily available iodoaniline **102** and propargyl substituted Schӧllkopf chiral auxiliary **103** [138] were coupled under Larock heteroannulation conditions regiospecifically to provide the 6-methoxyindole derivative **104** (Scheme 11). The Schӧllkopf chiral auxiliary severved as a protecting group which prevented epimerization of the chiral center in strong base. The required *N*_a_-methylation was executed with sodium hydride and iodomethane in dimethyl formamide to furnish the *N*_a_-methylated 6-methoxytryptophan derivative, in greater than 98% ee. The hydrolysis of the dihydropyrazine moiety with aqueous 2*N* HCl in THF and concomitant removal of the triethylsilyl group resulted in the formation of *N*_a_-methyl-6-methoxy-d-(−)-tryptophan ethyl ester **105** in excellent yield. The primary amine **105** was converted into the benzyl protected amine by reduction of the phenyl substituted imine with sodium borohydride, which had formed by condensation of benzaldehyde with the primary amine. The asymmetric Pictet-Spengler reaction of the so formed *N*_b_-benzyl protected tryptophan derivative with the aldehyde, ethyl 4-oxobutanoate under acidic conditions (HOAC in DCM) resulted in an almost quantitative yield of the di-ester **106** in a 72:28 (*trans*:*cis*) diastereomeric ratio. The same reaction in trifluoroacetic acid in DCM resulted in decomposition of the product. However, after the Pictet-Spengler reaction had been completed in acetic acid, the addition of a small amount of TFA was found to be effective for the epimerization of the diester at C-1 to the correscponding *trans*-diester isolated as a single diastereomer (>98% ee). The *trans*-diester **106** was subjected to a one-pot Dieckmann cyclization and this was followed by a base-mediated hydrolysis/decarboxylation process to provide the required 11-methoxy-*N*_b_-benzyl protected tetracyclic ketone **107** in excellent yield. The catalytic hydrogenation on palladium on carbon under acidic conditions, removed the benzyl group to provide the desired the *N*_b_-H tetracyclic ketone **108**, as shown on Scheme 11.

**Scheme 12:**
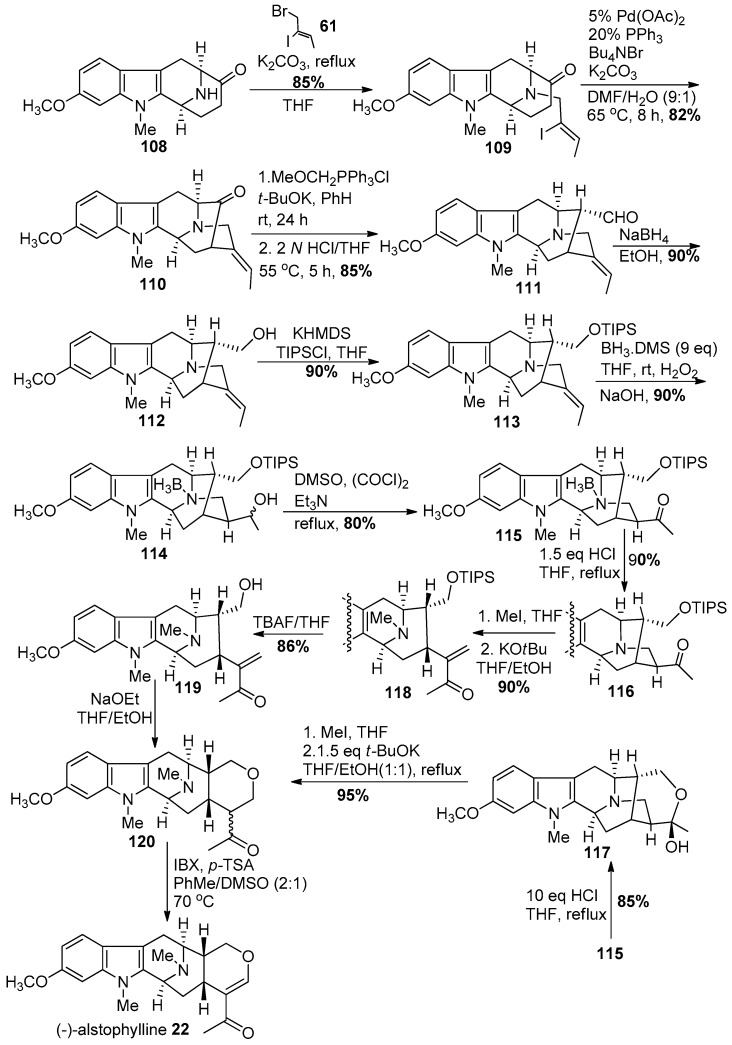
Enantiospecific total synthesis of (−)-**22** by Liu et al. [139].

The *N*_b_-H function in amine **108** was re-alkylated via an S_N_2 substitution with (*Z*)-1-bromo-2-iodo-2-butene **61** in the presence of K_2_CO_3_ in THF to provide the *N*_b_-alkylated ketone **109** in good yield (Scheme 12) analogous to previous work. The iodo-2-butene **61** can now be prepared in 3 steps in stereospecific fashion on 500 gram scale. A palladium-catalyzed enolate-driven intramolecular cross-coupling process resulted in the pentacyclic ketone **110** in stereospecific fashion. A Wittig olefination was followed by hydrolysis of the enol ether which had resulted to yield the one-carbon homologated aldehyde **111** in excellent yield. The reduction of the aldehyde to the primary alcohol **112**, to generate 11-methoxy affinisine was executed smoothly with sodium borohydride in ethanol. This constituted the first total synthesis of **112** (11-methoxy affinisine). Protection of the primary alcohol as a silyl ether **113** was done under standard conditions with triisopropylsilyl chloride in the presence of base. The hydroboration of the *E*-ethylidene function with borane-dimethyl sulfide and oxidation with basic hydrogen peroxide went smoothly to furnish the secondary alcohol **114**, accompanied by a small amount of the corresponding tertiary alcohol. The Swern-oxidation conditions were employed to convert the mixture of secondary alcohols into the corresponding ketone **115**. The *N*_b_-BH_3_ adduct which had formed, could be converted into the free amine **116** by treating it with 1.5 equivalents of aqueous HCl in refluxing THF. On the other hand, in the presence of 10 equivalents of HCl in refluxing THF the cyclic hemiacetal **117** (11-methoxy-*N*_a_-methyl-trinervine) was isolated in 85% yield, which could be converted into **120** by quaternization of the *N*_b_ nitrogen atom with iodomethane. This was followed by treatment with base to provide a *retro*-Michael reaction which underwent recyclization to dihydroalstophylline **120**.

Alternatively, the ketone **116** underwent the ring opening process under conditions similar to LeQuesne et al. [140] to **120**. The dihydroalstophylline upon treatment with IBX and *p*-TSA in toluene and DMSO gave (−)-alstophylline **22**, albeit the yield of this step was only moderate.

**Scheme 13:**
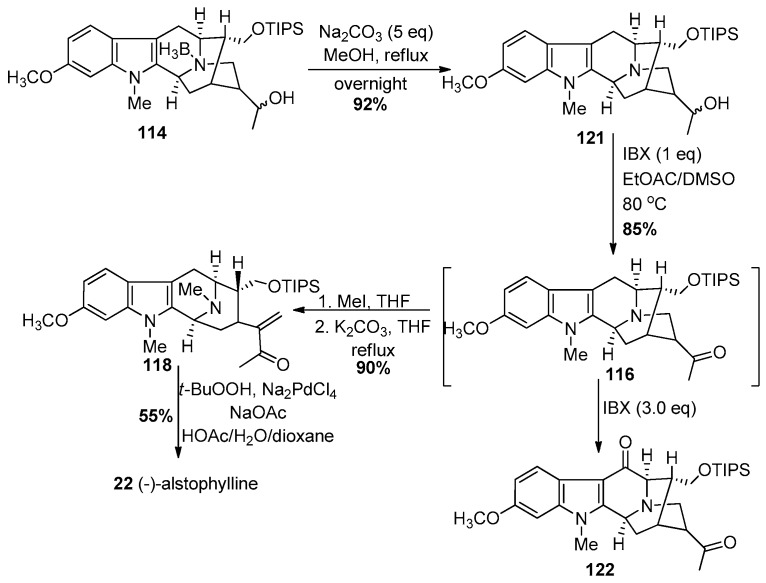
An improved synthesis of (−)-**22** by Liao et al. [141].

In an improved approach, Liao et al. in Milwaukee developed a modified Wacker reaction to gain quick access to several macroline-type indole alkaloids (Scheme 13) [141]. The *N*_b_-BH_3_ adduct **114** (Scheme 13) was accessed in similar fashion in better yield than the previous report by Liu et al. [139]. However, in this case, the *N*_b_-BH_3_ group was removed alternatively by refluxing the complex with sodium carbonate in methanol to provide **121**. The conditions of K.C. Nicolaou with IBX [142] were employed to oxidize the secondary alcohol to a ketone **116** (1 h) in 85% yield with the first equivalent of IBX. This was followed by addition of another 3 equivalents of IBX after the initial transformation and the mixture was heated to 80 °C. This promoted the radical process with IBX, as reported by Nicolaou et al. at the C-6 benzylic position to provide access to the 6-oxoalstophylline intermediate **122**. This method permitted access to both alstophylline and its 6-oxo variant simply by changing the number of equivalents of IBX. The ring opening of the ketone **116** by a *retro*-Michael reaction under conditions modified from those of LeQuesne et al. gave the *α*,*β*-unsaturated system in **118**. The ketone, under the modified Wacker conditions [143], effected the novel cyclization to provide (−)-alstophylline **22** directly. The total synthesis of alstophylline, accompanied by the total synthesis of (+)-macroline **19** by the group in Milwaukee constituted a total synthesis of (+)-macralstonine **9**. In addition, the unique indole alkaloid 6-oxoalstophylline was synthesized for the first time, as well as a much improved route to (−)-alstonerine via this modified Wacker (Pd (II)) process. This process can be employed to prepare a number of enone systems (**22b**, Figure 7) found in indole alkaloids, as well as flavones (Lorenz, Cook et al.).

### 4.4. (+)-Macralstonidine

(+)-Macralstonidine **8** represents another dimeric indole alkaloid which consists of a macroline and a sarpagine obligate monomeric unit. It has been isolated from *Alstonia macrophylla* [127,128], *Alstonia somersentenis* [127], and *Alstonia spectabilis* [133]. The alkaloid, *N*_a_-methylsarpagine (**23**) is a natural product isolated from *Alstonia spectabilis* by Hart et al. [133]. Zhao et al. reported the first total synthesis of (+)-*N*_a_-methylsarpagine, as well as the total synthesis of (+)-macralstonidine, along with several other indole alkaloids [144,145]. The synthesis of (+)-*N*_a_-methylsarpagine accompanied by the total synthesis of (+)-macroline *vide supra* by the group in Milwaukee [85,86] constituted a total synthesis of this bisindole alkaloid.

**Scheme 14:**
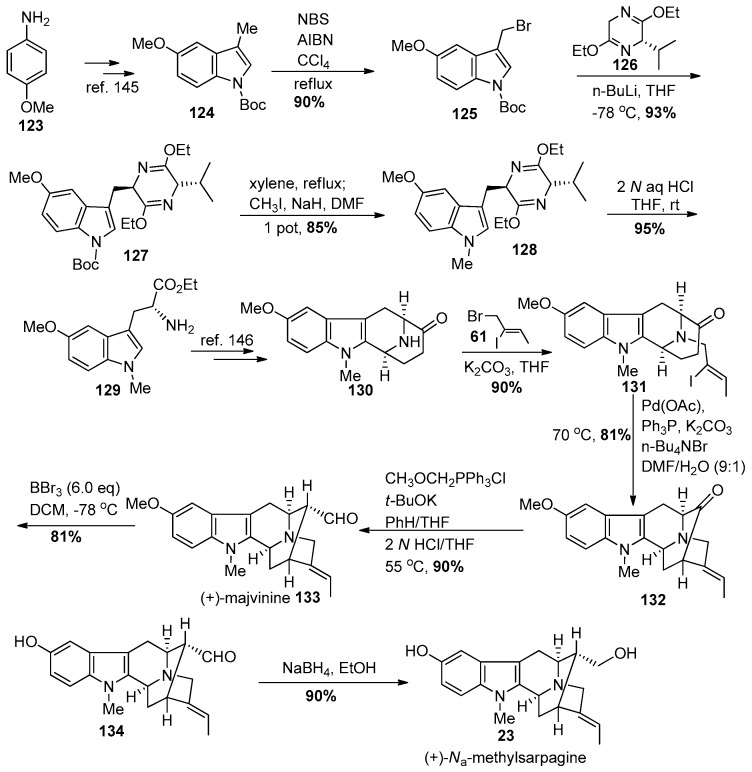
Enantiospecific total synthesis of (+)-**23** by Zhao et al. [144,145].

Synthesis of the desired sarpagine unit **23** began from *p*-anisidine **123** (Scheme 14) [145]. The 5-methoxy-3-methylindole-2-carboxylate was prepared on 600 gram scale via a Japp-Klingmann azo-ester intermediate [146]. The subsequent saponification was followed by a copper/quinoline-mediated decarboxylation process executed again on large scale in excellent yield. In this process only 2 equivalents of distilled quinoline were employed in contrast to the previous methods, which require a very large excess of quinoline. This latter route was difficult because the excess quinoline was hard to remove. The *N*_a_-H group was protected with a Boc function by treating it with Boc-anhydride and dimethyl amino pyridine to provide **124** in 95% yield. The regiospecific bromination of the C-3 alkyl group was done carefully under radical conditions with *N*-bromosuccinimide and AIBN (admixed and added in portions) in carbon tetrachloride on large scale and in excellent yield to furnish **125**. This radical reaction was also executed in refluxing cyclohexane with similar results. The 3-bromomethyl indole was found to be very unstable and was used without further purification. It was treated with the anion of the Schӧllkopf chiral auxiliary **126** at −78 °C to afford a single diastereomer **127** in 93% yield. The Boc group was removed under thermal conditions and this was followed by *N*_a_-methylation with iodomethane and sodium hydride in DMF to provide the *N*_a_-methyl tryptophan precursor **128**. The dihydropyrazine moiety present in the Schӧllkopf unit was hydrolyzed under acidic conditions in ethanol to furnish the 5-methoxy-*N*_a_-methyl d-(+)-tryptophan ethyl ester **129** on large scale. The usual subsequent transformations e.g., *N*_b_-benzylation, asymmetric Pictet-Spengler condensation, diastereospecific Dieckmann cyclization-decarboxylation reaction and catalytic debenzylation on palladium/carbon to remove the *N*_b_-benzyl group afforded the 5-methoxy tetracyclic ketone **130** in excellent overall yield. The *N*_b_-nitrogen atom was re-alkylated with the vinyl iodide, (*Z*)-1-bromo-2-iodo-2-butene **61** (prepared regiospecifically on 500 gram scale) to give iodo olefin **131**, which underwent the enolated-driven palladium-catalyzed cross-coupling to provide the *α*-vinylated intermediate, the key pentacyclic ketone **132** [144,145]. The conversion of the *E*-ethylidene intermediate **132** into the *α*-aldehyde [(+)-majvinine] **133** was effected using a one-carbon homologation sequence by Wittig olefination followed by hydrolysis/epimerization as employed previously in Milwaukee. The C-10 methoxy alkyl group was removed using 6.0 equivalents of boron tribromide in DCM at −78 °C to yield the 10-hydroxy intermediate **134** and this was followed by reduction of the aldehyde with sodium borohydride in ethanol to the primary alcohol in (+)-*N*_a_-methylsarpagine **23** in 90% yield over the two steps. This constituted the first enantiospecific total synthesis of (+)-majvinine and of (+)-*N*_a_-methylsarpagine **23** [144,145].

**Scheme 15:**
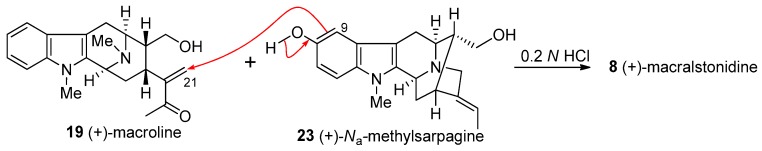
Synthesis of (+)-**8** by condensing (+)-macroline (**19**) and (+)-*N*_a_-methylsarpagine (**23**) under acidic conditions.

**Figure 9:**
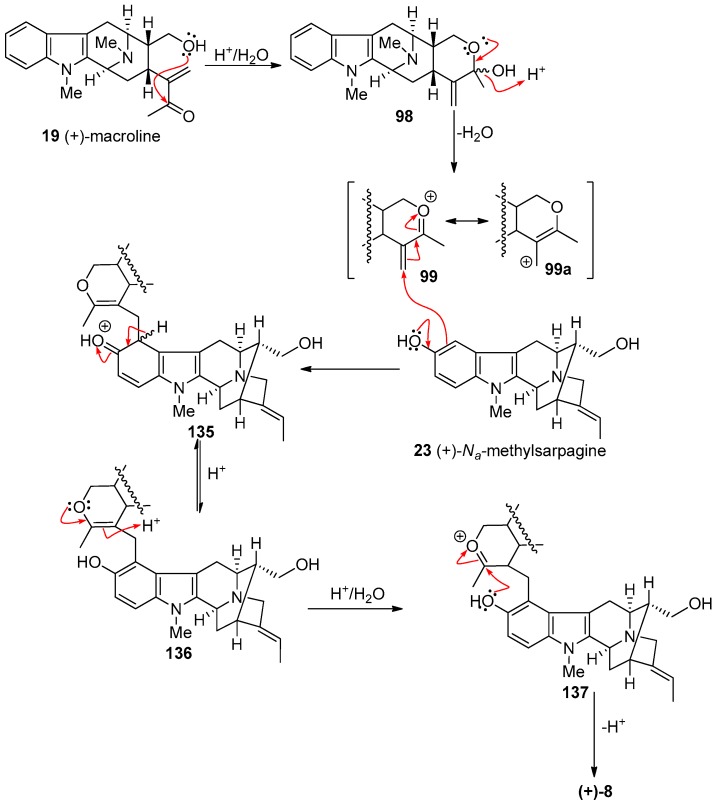
The possible alternative mechanism for the formation of (+)-**8** via a Friedel-Crafts alkylation process.

The condensation of (+)-macroline **19** and (+)-*N*_a_-methylsarpagine **23** under mildly acidicconditions provided the bisindole alkaloid, (+)-macralstonidine **8** (Scheme 15) [133]. In similar fashion to the case of macralstonine, there are at least two possible mechanisms of the coupling process. The first is the straight forward biomimetic coupling via a Michael reaction (Scheme 15) pioneered by LeQuesne [135], while the second is the Friedel-Crafts alkylation process [136,147], as illustrated in Figure 9. From a biological perspective the Michael reaction is the most reasonable, however, from a chemical perspective the Friedel-Crafts alkylation (Figure 9) is more likely. What is of significance here is that the alkylation took place soley at the C-9 position. No alkylation at C-11 was observed under these conditions.

### 4.5. (+)-Villalstonine

Villalstonine **5** is a bisindole alkaloid consisting of two monomeric indole alkaloid units and Schmid et al. concluded from their study of the degradation of (+)-villalstonine that the monomeric components were (+)-macroline **19** and (+)-pleiocarpamine **14** [148]. Villalstonine has been isolated from the leaves and stem bark of *Alstonia angustifolia* [131,149], *Alstonia macrophylla* [22,128,148], *Alstonia muelleriana* [77], and *Alstonia spectabilis* [127]. The structure was deduced by Schmid et al. and confirmed by Nordman et al. by X-ray crystallography [150]. (+)-Pleiocarpamine, one unit, has been isolated from many Apocynaceae plants. To date, it has been isolated from *Alstonia macrophylla* [22], *Alstonia angustifolia* [131], *Pleiocarpa mutica* [151], *Pleiocarpa pycnantha* [152], *Pleiocarpa tubicina* [151], *Kopsia dasyrachis* [152], and from *Hunteria eburnean* Pichon [153] as well as *Alstonia muelleriana* [73]. The structure was based on examination of the NMR spectra, as well as in depth studies via mass spectrometry [154].

LeQuesne et al. executed a biomimetic synthesis of (+)-villalstonine **5** from condensation of (+)-macroline with (+)-pleiocarpamine **14** [83]. Macroline was obtained by degradation of villamine (not shown) and stirred with (+)-pleiocarpamine **14** in 0.2 *N* aqueous hydrochloric acid solution at 20 °C for 18 h. This was followed by basic workup to furnish (+)-villalstonine **5** indistinguishable from the natural product [83].

**Scheme 16:**
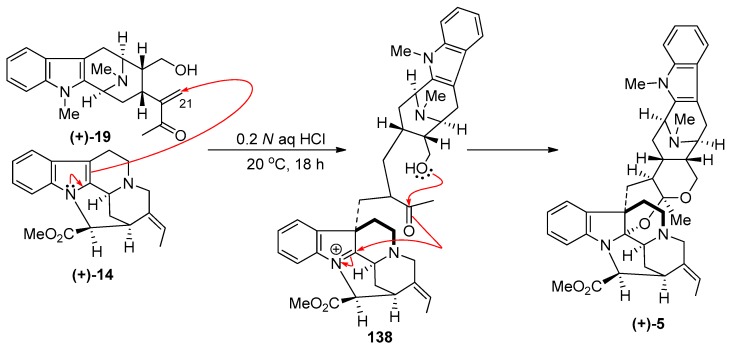
The biomimetic partial synthesis of (+)-**5** by LeQuesne et al. [83].

This biomimetic synthesis [83] of (+)-villalstonine is believed to consist of the initial electrophilic addition of the macroline enone **19** function onto pleiocarpamine **14** from the less hindered *α*-face to give intermediate **138**. This intermediate undergoes facile transformation via hemiacetal formation and subsequent or concerted attack on the iminium ion of the indoline **138** to form the acetal-aminoactetal function in (+)-villalstonine **5**, as illustrated in Scheme 16.

**Scheme 17:**
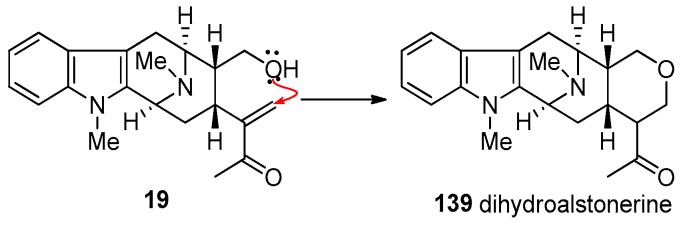
Intramolecular cyclization of macroline (**19**) to form **139** which is responsible for its limited shelf life.

Since the total synthesis of (+)-pleiocarpamine **14** has not been successfully executed to date, the total synthesis of (+)-villalstonine **5** has still not been reported. In a partial synthesis of villalstonine, the Milwaukee group employed the more stable macroline equivalent **140**, which served as a better alternative than macroline **19** itself, for the condensation with another monomeric unit to access bisindole alkaloids. This is because (+)-macroline tended to cyclize to form dihydroalstonerine **139** and limited the shelf life of macroline in storage (Scheme 17) [86].

**Scheme 18:**
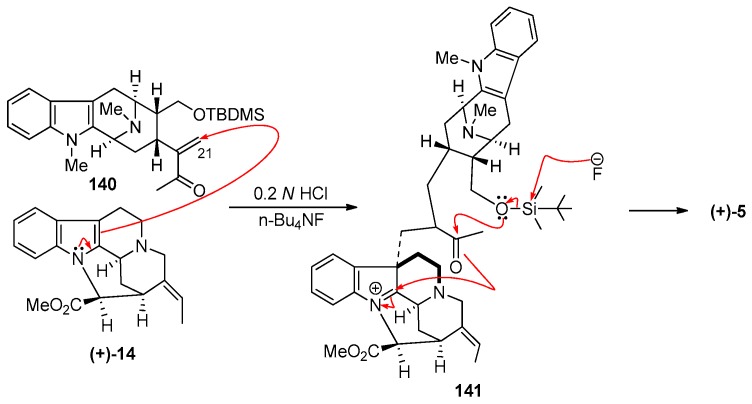
An improved partial synthesis of (+)-villalstonine (**5**) using the stable macroline equivalent (**140**) by Bi et al. [86].

The stable macroline equivalent **140** was stirred with natural (+)-pleiocarpamine **14**, obtained from Professor LeQuesne, in 0.2 *N* aqueous hydrochloric acid along with tetrabutylammonium fluoride at 20 °C for 24 h. (+)-Villalstonine **5** was obtained as the sole product and was indistinguishable from the natural (+)-villalstonine. It has not been confirmed whether the desilylation of the macroline equivalent **140** occurred to form macroline **19** before the condensation or the condensation took place similar to that depicted in Scheme 18. It is significant that the stable dihydroalstonerine **139** was **not** obtained in this process.

### 4.6. (−)-Macrocarpamine

**Scheme 19:**
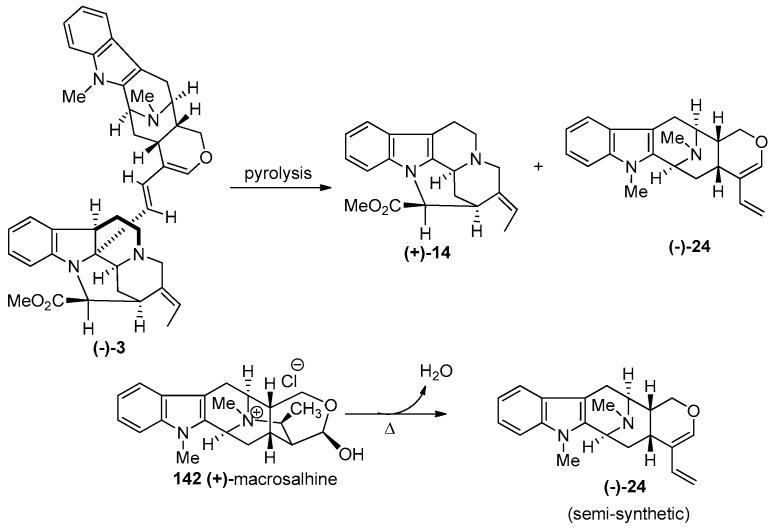
Semi-synthetic access to (+)-**14** and (−)-**24** by the pyrolysis of (−)-macrocarpamine (**3**) and (+)-macrosalhine (**142**).

(−)-Macrocarpamine **3** is a bisindole arising from the condensation of (+)-pleiocarpamine **14** and the indole base (−)-anhydromacrosalhine-methine **24**, according to the biogenetic pathway proposed by Hesse et al. [155]. It has been found in *Alstonia macrophylla* WALL [40,155], and *Alstonia angustifolia* [21,156]. (−)-Anhydromacrosalhine-methine **24** was first reported as a degradation product of (+)-macrosalhine **142** [157], as well as (−)-macrocarpamine **3** [155] (Scheme 19). The partial synthesis of macrocarpamine was carried out by Wang et al. from the condensation of natural (+)-pleiocarpamine **14** and semi-synthetic (see Scheme 19) (−)-anhydromacrosalhine-methine **24** [158,159,160].

**Scheme 20:**
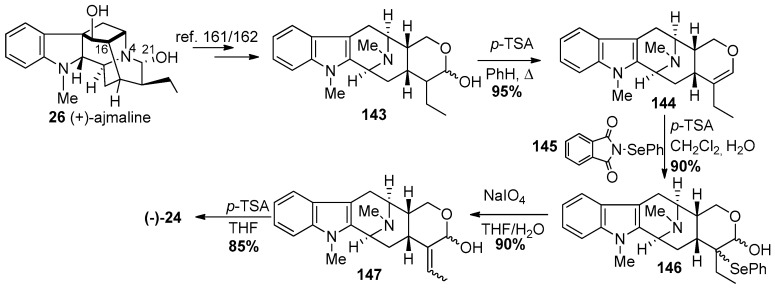
Semi-synthetic access to (−)-**24** from relay compound (+)-ajmaline (**26**) [160].

In the partial synthesis of (−)-macrocarpaine **3**, an authentic sample of (−)-anhydromacrosalhine-methine **24** (a relay compound) was first prepared semi-synthetically from natural (+)-ajmaline **26** (Scheme 20) [160]. The hemiacetal **143** was prepared from ajmaline **26**, according to the reported procedure by Sakai [161] and also carried out by Wearing et al. [162] which involved generation of the indole from the indoline, epimerization at C-16 and cleavage of *N*_b_-C21 bond. This yielded the E ring in macrosalhine methine **24**. In this sequence the hemiacetal **143** underwent a dehydration to form deoxyalstonerine **144** upon heating with *p*-toluenesulfonic acid in refluxing benzene. Oxyselenation of **144** to provide **146** was carried out using *N*-(phenylseleno)phthalimide in CH_2_Cl_2_ in the presence of 2-3 equivalents of water and 1.3 equivalents of *p*-toluenesulfonic acid. Elimination of the selenoxide upon treatment of **146** with NaIO_4_ provided the allylic alcohol **147**. The 1,4-elimination in **147** of water was executed by stirring it with *p-*toluenesulfonic acid in THF to provide (−)-anhydromacrosalhine-methine **24** in 85% yield [160].

**Scheme 21:**
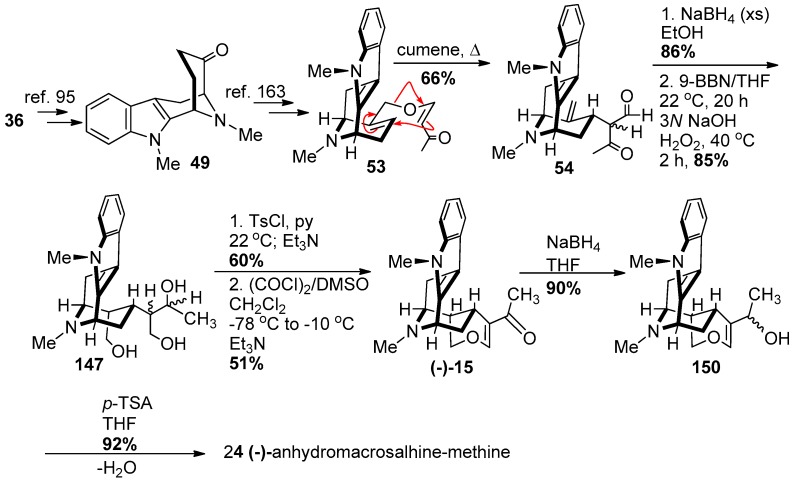
Enantiospecific total synthesis of (−)-**24** by Gan et al. [158].

The enantiospecific total synthesis of (−)-anhydromacrosalhine-methine **24** began from d-(+)-tryptophan. The tetracyclic ketone **49** was prepared in >98% ee using the reported method (Scheme 21) [95] on large scale. (−)-Alstonerine **15** which was converted into (−)-**24**, was prepared from **49** following the same procedure reported for the total synthesis of alstonerine **15** by Bi et al. [86]. The enone ether **53** was synthesized from the *N*_b_-methyl tetracyclic ketone **49** in four steps following the reported procedure [163] as described in brief here. The Claisen rearrangement of enone **53** took place from the desired *α*-face with at least 4:1 diastereoselectivity to provide **54** in 66% yield. Reduction of the carbonyl functions with sodium borohydride in ethanol was followed by stereospecific hydroboration-oxidation of the olefin with 9-BBN/THF and H_2_O_2_/NaOH which furnished the triol **147**. The regioselective cyclization of **147** by using the tosylate as a leaving group generated the tetrahydropyran which was subsequently oxidized to the enone **15** [(−)-alstonerine] in 51% yield. The enone was accompanied by 31% of dihydroalstonerine **139** which could be transformed into the enone **15** through reduction and re-oxidation. Reduction of (−)-alstonerine **15** with sodium borohydride to the allylic alcohol **150** was followed by dehydration using *p*-TSA in excellent yield to complete the enantiospecific total synthesis of (−)-anhydromacrosalhine-methine **24**.

**Scheme 22:**
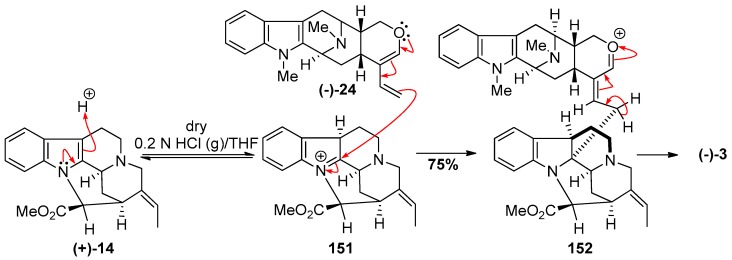
Partial synthesis of (−)-macrocarpamine (**3**) by Gan et al. [159].

When (−)-anhydromacrosalhine-methine **24** was condensed under mildly aqueous acidic conditions (0.2 *N* aqueous HCl) with natural pleiocarpamine **14**, the enol ether was converted into its hydrated form preventing it from undergoing the coupling reaction to yield (−)-macrocarpamine **3** [159]. However, under anhydrous conditions (0.2 *N* HCl (g)/THF), when 6 equivalents of **24** were added in portions over 48 h to (+)-pleiocarpamine, (−)-macrocarpamine **3** was formed in 75% yield (Scheme 22). A possible mechanism for the coupling reaction has been proposed by Wang et al. [159]. The proton acted as an electrophile to form the readily reversible iminium ion **151**. The diene **24** was added portionwise in acidic solution to immediately quench the iminium ion upon its formation and to prevent the decomposition of the diene. The so formed (−)-macrocarpamine **3** was identical to the natural product reported by Hesse et al. by comparison of the spectral data [155].

### 4.7. (+)-Dispegatrine

(+)-Dispegatrine **7** is a dimeric indole alkaloid isolated from the water-soluble fraction of the root extract of *Rauwolfia verticillata* (Lour.) Bail. *var. hainanensis* Tsiang [164,165]. It is a bisphenolic as well as a bisquaternary sarpagine indole alkaloid which makes it a unique alkaloid since the occurrence of these types of alkaloids are very rare [18]. (−)-Blumeanine (not shown) [166] is the only other alkaloid of this class among more than 300 or so bisindole alkaloids isolated to date. The monomer, spagatrine **16** is a major alkaloid isolated from *Aspidosperma spegazzinii* [167], *R. sprucei* [168], *R. verticillata* var. hainanesis [164], and from *R. verticillata var. rubrocarpa* [165].

**Scheme 23:**
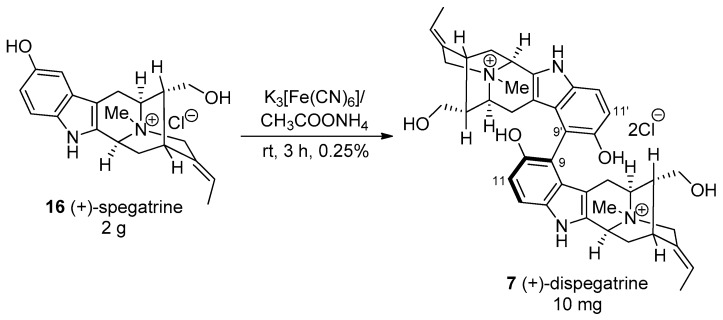
Biomimetic partial synthesis of dispegatrine by Yu et al. [164].

A biomimetic partial synthesis of (+)-dispegatrine **7** had been carried out from the monomer spegatrine **16** by Yu et al. [164] via an oxidative phenolic coupling in a negligible yield of 0.25% (Scheme 23). Although most of the structure of (+)-dispegatrine was determined based on the analysis of the NMR spectra and mass spectra, the partial synthesis by Yu confirmed the structure [164]. Moreover, no other diastereomer was reported. However, the isolation chemists were unable to determine the important axial chirality at the C9–C9′ bond [164]. In spite of that, formation of only one atropodiastereomer in that dehydrodimerization process suggested internal chiral induction was responsible for the atroposelectivity at the C9–C9′ bond. This strategy was employed in the total synthesis of (+)-dispegatrine **7** and its monomer, (+)-spegatrine **16** in a convergent fashion, by Edwankar et al. [169,170].

In a biomimetic total synthesis of the (*P*)-atropodiestereomer of (+)-dispegatrine [169,170], the tetracyclic ketone core **163** was accessed via the general process for ring-A oxygenated indole alkaloids, which began from 5-methoxy-d-(+)-tryptophan via the asymmetric Pictet-Spengler condensation and diastereospecific Dieckmann cyclization process.

**Scheme 24:**
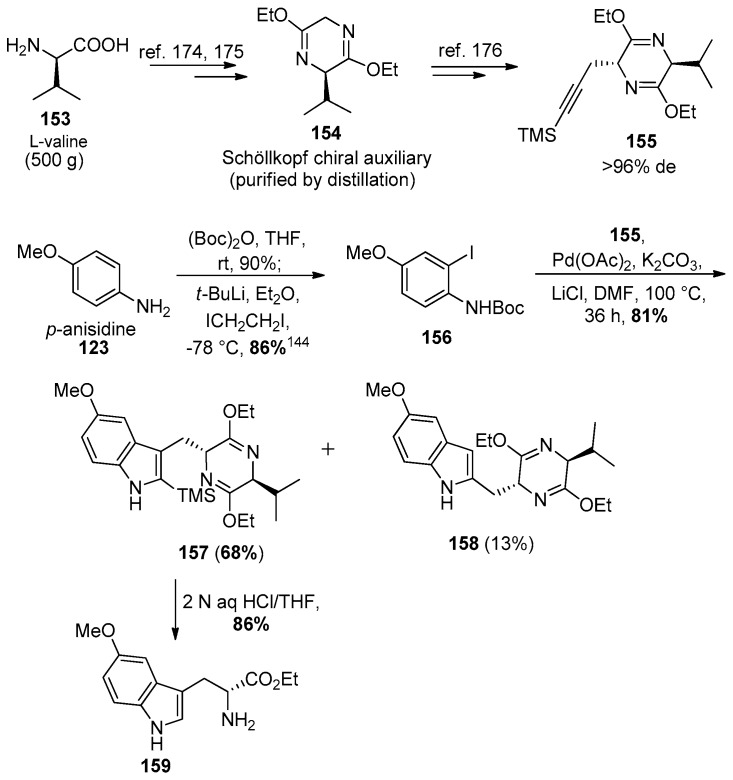
Enantiospecific access to 5-methoxy tryptophan (**159**) by The Larock heteroannulation process.

The enatiospecific synthesis of 5-methoxy tryptophan was done on large scale using a regiospecific bromination [145] and Larock heteroannulation [171,172,173] process. The l-valine in the Schöllkopf chiral auxiliary served both as the starting material and the source of chirality in the route. The Schöllkopf chiral auxiliary **154** was prepared on large scale (500 g) from l-valine **153** and glycine ethyl ester using an earlier method (Scheme 24) [174,175,176].

The *o*-iodoaniline **156** was synthesized by employing known methods (Scheme 24) [177,178]. The amino group in **123** was protected with a Boc group and subjected to a Snieckus *ortho*-lithiation [179] with 2.2 equivalents of *tert*-butyllithium at −78 °C and quenched with diiodoethane to afford the Boc protected iodoaniline **156** in 86% yield, required for the Larock heteroannulation. With the TMS-substituted alkyne **155** and iodoaniline **156** in hand, the heteroannulation was carried out using conditions developed by Ma et al. [176] which furnished the Schöllkopf analogs **157** and **158** in a combined yield of 81%. Using a bulkier silyl TES-substituted alkyne did not improve the regioselectivity of the cyclization. Hydrolysis of indole **157** under acidic conditions removed the Schöllkopf unit, as well as the silyl group to provide optically active 5-methoxy-D-tryptophan ethyl ester **159** in 86% yield.

**Scheme 25:**
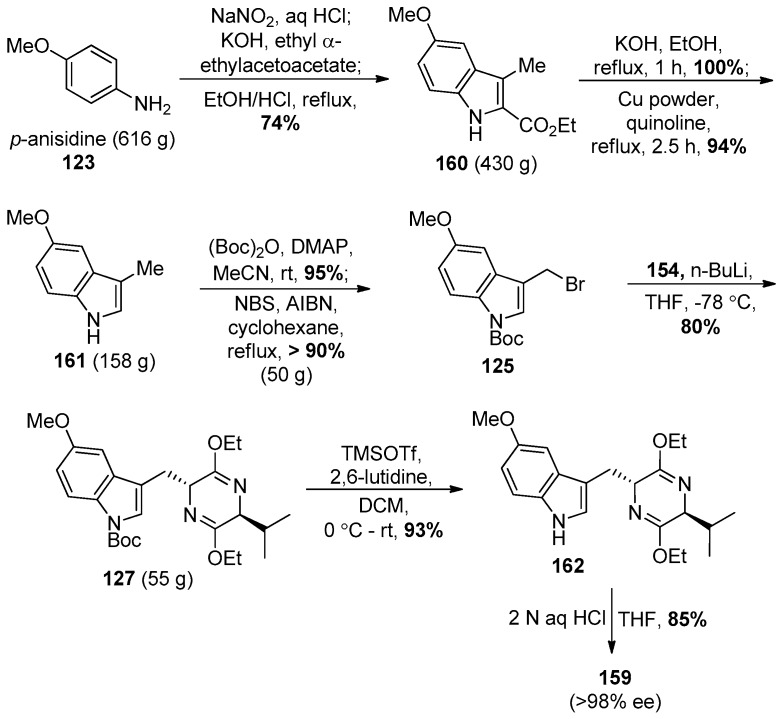
Alternative approach toward enantiopure **159** via the Japp-Klingemann/Fischer esterification process.

Due to the failure to improve the diastereoselectivity of the Larock heteroannulation (68:13) using the TMS-substituted alkyne **155** and the *N*-Boc protected *ortho*-iodo aniline **156** under the previously reported conditions [90,180], an alternative route involving a Japp-Klingemann/Fischer indole processes was also employed. This was developed by Abramovitch and Shapiro and was used here for the large-scale (>600 g) preparation of 3-methyl-5-methoxyindole **161** (Scheme 25) [181,182].

As mentioned above, the 5-methoxy-3-methylindole-2-carboxylate **160** was prepared on large scale via The Japp-Klingemann azo-ester intermediate (Scheme 25) [146]. The subsequent saponification was followed by a copper/quinoline-mediated decarboxylation executed on large scale to provide **161** in excellent yield. In order to carry out this decarboxylation in high yield only 2 equivalents of distilled quinoline were employed. The indole *N*-H group was then protected with a Boc group by treatment of **161** with Boc-anhydride and 4-(dimethylamino) pyridine in 95% yield. The subsequent regiospecific bromination of the C-3 alkyl group was done under radical conditions with *N*-bromosuccinimide and AIBN (admixed) in carbon tetrachloride on large scale in excellent yield to furnish bromide **125**. This radical reaction could be carried out in refluxing cyclohexane with the same result. Alkylation of bromide **125** with the anion of the Schöllkopf chiral auxiliary (**154**) provided **127** on multigram scale. An improved and milder condition for the removal of the Boc group using TMSOTf/2,6-lutidine [183] was used in place of refluxing xylene [145]. The hydrolysis of the Schöllkopf analog **162** with 2 *N* aqueous HCl in THF provided the optically active *N*_a_-H, 5-methoxy-d-(−)-tryptophan ethyl ester (**159**) in excellent yield and diastereoselectivity (>98% ee).

**Scheme 26:**
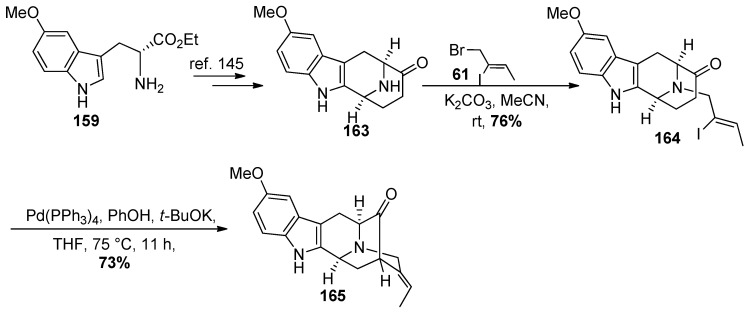
Synthesis of the 10-methoxy pentacyclic core system **165** from **159**.

The tetracyclic ketone **163** was prepared on large scale from ester **159** which involved the asymmetric Pictet-Spengler reaction [37,145,184,185,186], and diastereospecific Dieckmann cyclization [111]. This was followed by base mediated decarboxylation and catalytic debenzylation of the *N*_b_-benzyl function under acidic conditions (Scheme 26).

The *N*_b_-alkylation was carried out by heating (*Z*)-1-bromo-2-iodo-2-butene **61** [187] with **163** in the presence of potassium carbonate in MeCN to provide the desired vinyl iodide **164** (Scheme 26) [111,145]. Because this route to **164** was somewhat longer, the Larock heteroannulation was often employed to prepare 5-methoxy-d-(+)-tryptophan. The enolate mediate palladium-catalyzed intramolecular cross-coupling of the vinyl iodide **164** was carried out under the conditions of Bonjoch [188] which employed PhOK/Pd(PPh_3_)_4_ to effect the coupling in 73% yield (Scheme 26). Initial attempts with the modified conditions (*t*-BuOK only) resulted in the pentacyclic ketone **165** in only 50% yield [87]. This was because the acetylene by product was formed in the presence of the stronger base *t-*BuOK versus the Bonjoch sodium phenoxide.

**Scheme 27:**
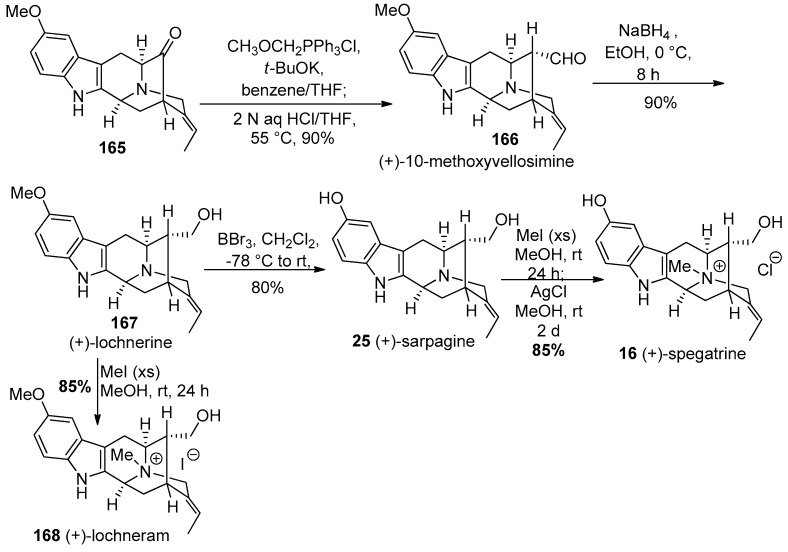
Enantiospecific total synthesis of (+)-10-methoxyvellosimine (**166**), (+)-lochnerine (**167**), (+)-sarpagine (**25**), (+)-spegatrine (**16**), and (+)-lochneram (**168**) by Edwankar et al. [169,170].

The *E*-ethylidene pentacyclic ketone **165** was subjected to a one-carbon homologation via a Wittig olefination- hydrolysis process employed many times in Milwaukee in a one pot sequence. This provided the desired *α*-aldehyde, the natural product (+)-10-methoxyvellosimine **166**, in excellent yield (Scheme 27). The reduction of **166** with sodium borohydride yielded another natural product, (+)-lochnerine (**167**). Upon treatment with BBr_3_ in methylene chloride at −78 °C, demethylation of **167** occurred in 80% yield to furnish the hydroxyindole natural product (+)-sarpagine **25**. The quaternization of **25** at the *N*_b_-function using an excess of iodomethane was followed by simple iodide halide exchange with silver chloride in ethanol to furnish (+)-spegatrine chloride (**16**) [169,170]. In addition to (+)-lochnerine, when it was treated with an excess of iodomethane in methanol at rt for 24 h this provided the natural product (+)-lochneram **168** in 85% yield. These monomeric indole alkaloids were synthesized for the first time, the chemical and physical properties of which were very important in the final assault on (+)-dispegatrine. One could determine the chemical and chromatography characeteristics of these monomers to help with the isolation and purification of (+)-dispegatrine.

**Scheme 28:**
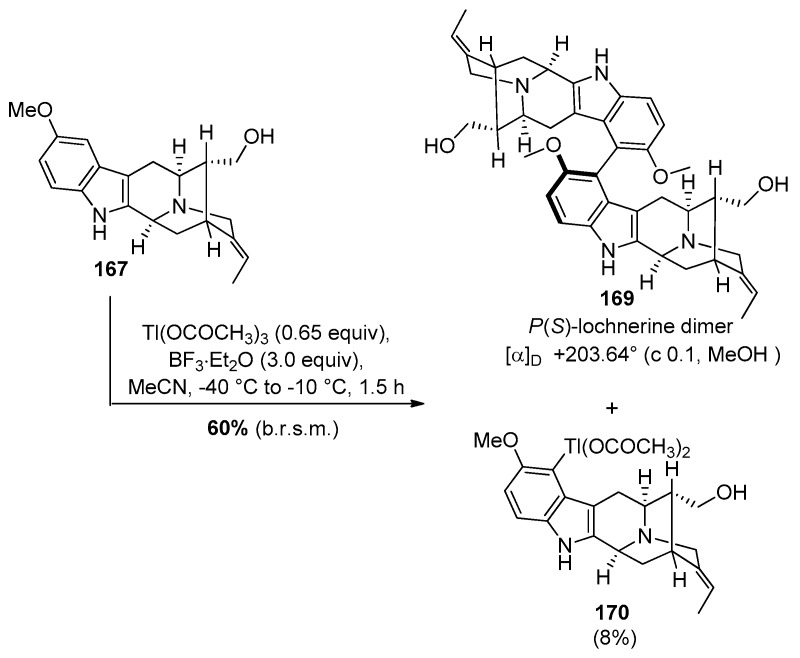
Thallium (III) mediated oxidative dimerization process to provide *P*(S)-atropodiastereomer of the dimer lochnerine (**169**).

Numerous attempts were undertaken (including Ullmann coupling and Suzuki-Miyaura coupling) to effect the biaryl cross-coupling in model substrates while avoiding the very poor yields in the oxidative phenolic coupling reported in the partial synthesis of (+)-dispegatrine **7** by Yu et al. [170]. Eventually, a thallium(III) mediated Scholl-type dimerization [189] of (+)-lochnerine **167** was successfully executed wherein the chirality of the C9–C9′ bond was determined by the chirality inherent in the sarpagine system, as planned (Scheme 28).

As shown in Scheme 28, The monomeric sarpagine alkaloid lochnerine **167**, under the modified conditions of Taylor et al. [190,191] underwent Tl(III) mediated oxidative dimerization (a 0.65 equvalent of thallium (III) acetate with 3 equivalents of borontrifluoride etherate in MeCN at −40 °C) to furnish the key C9–C9′ biaryl coupling and provided the *P*(S)-atropodiastereomer **169** (confirmed by X-ray crystallographic analysis at low temperature wherein all the chiral centers were determined with the MoKα source) as the sole product and with complete regioselectivity [169]. This process was accompanied by a competing byproduct, an electrophilic thallation product **170** and its origin could be understood by the mechanistic studies of Kochi et al. [192,193].

**Scheme 29:**
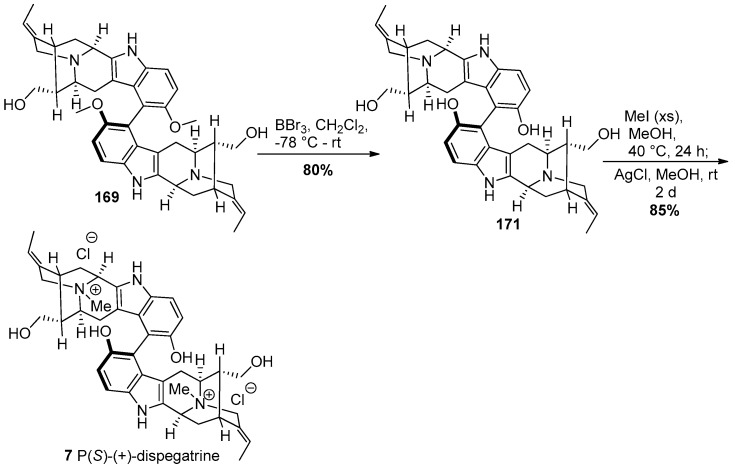
Completion of the first total synthesis of *P*(S)-(+)-dispegatrine (**7**) by Edwankar et al. [170].

The methoxy group in **169** was demethylated using an excess of boron tribromide in methylene chloride at −78 °C to provide the sarpagine dimer **171** followed by *N*_b_-quaternization using excess iodomethane in methanol at 40 °C to provide the dispegatrine diiodide salt (Scheme 29). Treatment of the diiodide salt with silver chloride in methanol provided (+)-dispegatrine **7** in 85% yield. Although the (*P*)-atropoisomer here could be correlated back to the configuration in the *P*(S)-(+)-lochnerine dimer, the stereochemistry was confirmed by examination of extensive NMR studies. This strategy completed the first total synthesis of the monomer spegatrine as well as the dimer *P*(S)-(+)-dispegatrine in a nature inspired and convergent manner in 8.3% yield starting from commercially available material. Moreover 6 other monomeric 10-methoxy substituted indole alkaloids were synthesized, enantiospecifically, for the first time.

## 5. Conclusions

Indole alkaloids of different classes and sub-classes have been a promising source of new drugs since the search for new molecular entities with profound biological activity from natural sources was initiated. It is sure to continue. The pace of natural product-based drug development from plants to patients has necessarily been rather moderate because of the difficulty in isolation of sufficient material for adequate structure determination, initial screening for a target, followed by preclinical and clinical studies. The anticancer properties of the important bisindole alkaloids, vincristine and vinblastine, prompted chemists including Potier, Kutney, Kuehne, Magnus, Rawal, and Fukuyama to execute elegant work in this area. In addition, natural product derivatives are an equally important area of further improvements in drug development. In the case of bisindoles, the bisindole itself continues to be more potent in most cases in comparison to the monomeric alkaloids which comprise them. It is this phenomenon that stimulated the interest in the synthesis of the antimalarial and antileishmanial bisindoles described herein. In order to accelerate the development of the drugs from natural sources, modern, practical, and efficient synthetic strategies capable of scale up to multigram quantities are of utmost importance. Illustrated in this review is the versatility of the asymmetric Pictet-Spengler reaction in the enantiospecific total synthesis of many biologically active monomeric and bisindole alkaloids. In addition, there are many other new developments with 100% diastereoselectivity derived from the coupling process of two monomeric alkaloids to provide the bisindole targets. Investigations of the synthesis of the monomeric unit has yielded many reactions capable of scale up, to date, to the 300–600 g levels.
